# *Thymus apulus* (*T.* sect. *Hyphodromi*, Lamiaceae), a New Species from Southern Italy

**DOI:** 10.3390/plants14233584

**Published:** 2025-11-24

**Authors:** Fabrizio Bartolucci, Fabio Conti

**Affiliations:** Floristic Research Center of the Apennine, University of Camerino—Gran Sasso Laga National Park, San Colombo, 67021 Barisciano, Italy

**Keywords:** Alta Murgia National Park, endemic, Mediterranean flora, morphometry, Puglia, taxonomy

## Abstract

*Thymus apulus*, a new species from the calcareous highland (Murge hill area) of Apulia and Basilicata, southern Italy, is described and illustrated. The new species belongs to *Thymus* sect. *Hyphodromi*, and has been confused in the past with *T. striatus* (a southeastern European species), or *T. spinulosus* (a species strictly endemic to central and southern Italy). A morphometric analysis to assess the taxonomic relationships of the putative new species and the closely related *T. spinulosus* and *T. striatus* was carried out. Multivariate and univariate morphometric analyses demonstrate that *T. apulus* is clearly different from *T. striatus* and *T. spinulosus*. It can be reliably identified by a combination of quantitative and qualitative diagnostic characters, including pink corolla, capituliform to elongate inflorescence, predominantly glabrous leaves with rare sessile oil glands and non-parallel lateral veins, and distinct morphometric traits of the bracts and calyx. The distribution of *T. apulus* is restricted to several localities in the Murge highland (Apulia and Basilicata), within Alta Murgia National Park (SCI IT9120007 “Alta Murgia”) and Murgia Materana Park (SCI IT9220135 “Gravine di Matera”). *Thymus apulus* usually grows in sub-Mediterranean xeric grasslands corresponding to habitat 62A0, “Eastern sub-Mediterranean dry grasslands (*Scorzoneratalia villosae*)”, included in Annex I of the Habitats Directive (92/43/EEC). Furthermore, the conservation status assessment of the new species, according to IUCN categories and criteria, is proposed and discussed, and an analytical key for dried herbarium specimens to the species of *T.* sect. *Hyphodromi* in Italy is presented.

## 1. Introduction

*Thymus* L. is regarded as one of the largest genera in the *Lamiaceae* family, encompassing important medicinal and aromatic plants. It is one of the most emblematic genera belonging to taxonomically complex groups, characterized by intricate evolutionary relationships, hybridization, polyploidy, high phenotypic variability with unclear boundaries between species, and a nomenclature nightmare [1,2,3]. *Thymus* consists of about 270 accepted species occurring as native across Europe, NW Africa, Ethiopia, Asia, and Greenland [4,5].

In Italy, 21 species and subspecies are currently recognized, of which only 5 are considered endemic [6,7,8]: *T. spinulosus* Ten. (central and southern Italy), *T. richardii* subsp. *nitidus* (Guss.) Jalas (Sicily), *T. picentinus* (Lacaita) Bartolucci (southern Italy), *T. paronychioides* Čelak. (Sicily), and *T. praecox* subsp. *parvulus* (Lojac.) Bartolucci, Peruzzi & N.G.Passal. (Sicily).

During floristic investigations carried out in May 2009 in Apulia (southern Italy), an enigmatic and morphologically peculiar population of *Thymus* was discovered. This population did not correspond to any known species present in Italy or, more broadly, in the southeastern Euro-Mediterranean region [8,9,10,11,12,13,14,15,16,17,18]. This *Thymus* was collected in the Alta Murgia (central Apulia), which constitutes the north-western part of the calcareous highlands of the Murge hill area, and in the Murgia Materana (Basilicata), which can be regarded as an extension of the Apulian Murge. These areas are included respectively within Alta Murgia National Park and Murgia Materana Park (also known as Parco Archeologico Storico Naturale delle Chiese Rupestri del Materano). We visited the same area in June 2016 to collect material for studying the chemical composition and biological activity of select *Thymus taxa* [19]. Thanks to a specific project conceived and developed in synergy with Alta Murgia National Park, which also funded it, specific field investigations were undertaken in May 2025 with the specific aim to collect living and dried specimens for a taxonomic study, as well as to investigate its distribution and ecology. The undescribed *Thymus* species coexists with *T. spinulosus* but flowers at a different period and is clearly morphologically distinct. It exhibits, similar to *T. spinulosus*, non-leaf-like bracts, a key morphological trait that places it within *T.* sect. *Hyphodromi* (A. Kern.) Halácsy.

*Thymus* sect. *Hyphodromi* is distributed in the Mediterranean region and in the Caucasus to Central Asia and includes approximately 70 species [4,10,11,12,15,16,17,20,21,22,23,24,25,26,27,28,29]. Species belonging to *T.* sect. *Hyphodromi* are characterized by holotrichous or amphitrichous stems, flat leaves or leaves with revolute margins, capituliform to interrupted inflorescences, and usually not leaf-like bracts. Three subsections [30] are currently recognized within this section but only two are reported from Italy [8,21]: *T.* sect. *Hyphodromi* subsect. *Subbracteati* (Klokov & Des.-Shost.) Jalas, distributed in central Spain, northern Africa, south-eastern Europe, and Western Asia, characterized by leaves linear to elliptical, rarely narrowly spathulate, with parallel venation, calyx campanulate, and usually not leaf-like bracts; *T.* sect. *Hyphodromi* subsect. *Serpyllastrum* Villar, distributed in Spain, Sicily, Balkan Peninsula, Crimea, Turkey, the Caucasus, and Central Asia, is characterized by spathulate to obovate leaves with non-parallel venation, a cylindric-campanulate calyx, and bracts that are similar to the cauline leaves or not leaf-like [20,30].

Within *T.* sect. *Hyphodromi*, only three species are currently reported for the Italian flora [7,21,22]: *T. paronychioides* Čelak. (*T.* subsect. *Serpyllastrum*), endemic to Sicily; the southeastern European *T. striatus* Vahl (*T.* subsect. *Subbracteati*), distributed mainly along the Apennines with two subspecies (subsp. *striatus* and subsp. *acicularis* (Waldst. & Kit.) Ronniger); and *T. spinulosus* Ten. (*T.* subsect. *Subbracteati*), endemic to central and southern Italy.

A third peculiar *taxon* belonging to *T. striatus*, is subsp. *interruptus* (Jalas) Bartolucci, distributed only in the Thracian plain (European Turkey and south Bulgaria) at the eastern limit of the range of the species [3,11,15,31]. Other species within this section are reported from the Balkan Peninsula, whose flora often has similarities with the flora of central and southern Italy; however, all exhibit morphological differences from the *Thymus* population identified in the Murge hill area: *T. atticus* Čelak., *T. aznavourii* Velen. *T. boissieri* Halácsy, *T. bracteosus* Vis. ex Benth., *T. comptus* Friv. *T. dolopicus* Formánek, *T. holosericeus* Čelak., *T. jalasianus* Stoyanov & Marinov, *T. laconicus* Jalas, *T. leucotrichus* Halácsy, *T. parnassicus* Halácsy, *T. perinicus* (Velen.) Jalas, *T. plasonii* Adamović, and *T. zygioides* Griseb [3,9,10,12,13,14,15,16,17,18,23,29,32,33].

The *Thymus* population of the Murge area has previously been confused with *T. striatus*, a species recently excluded from the flora of Apulia [21], or with *T. spinulosus*, a species that is quite common in southern Italy [8,21]. It was first reported by Palanza [34], who clearly distinguished two *Thymus taxa* in the Murge area, both morphologically constant and uniform, with no intermediate forms: one under the name *T. zygis* L. (a misapplied name by Italian authors in the past, referring to the *T. striatus* species complex), with purple flowers (“*floribus purpureis*”) and a calyx with rare glands (“*Calycibus … parce glandulosis*”), and one with white flowers (“*floribus albis*”) and a calyx with dense glands (“*Calycibus glandulosis*”), described as *T. zygis* var. *virescens* Guss. (referring to *T. spinulosus*). Later, several authors reported for that area two *taxa* within *T.* sect. *Hyphodromi*, citing usually only *T. spinulosus* [35,36,37,38,39,40,41], and occasionally both *T. spinulosus* and *T. striatus* [42,43,44].

The present study, which is part of the ongoing taxonomic revision of the genus *Thymus* in Italy and the Euro-Mediterranean region [3,8,21,22,45], aims to clarify the taxonomy, chorology, and ecology of the newly described species *T. apulus*, through comparative morphological analyses with the closely related taxa *T. striatus* and *T. spinulosus*.

## 2. Materials and Methods

### 2.1. Sampling

**Figure 1:**
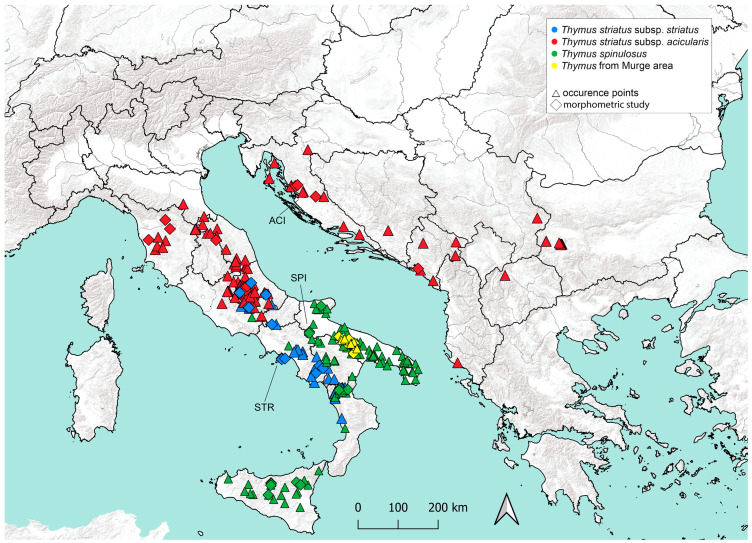
Distribution of studied populations for morphometric analyses (diamonds) and occurrence points (triangles) of *Thymus striatus* subsp. *striatus* (STR, blue diamonds and triangles), *T. striatus* subsp. *acicularis* (ACI, red diamonds and triangles), *T. spinulosus* (SPI, green diamonds and triangles), and *Thymus* from the Murge area (APU, yellow diamonds and triangles) in south-eastern Europe. The type localities of STR, ACI, and SPI are indicated by a line.

A total of 955 herbarium samples kept at APP, AQUI, BI, BM, BRNU, CAME, CAT, CLU, FI, G, HFLA, IS, IT, LEC, NAP, P, PAL, PI, PORUN, PR, RO, W, WU, and ZA public herbaria (Supplementary Material File S1; codes follow [46]), as well as in the private collections of L. Cancellieri (*Herb. Cancellieri*), R.P. Wagensommer (*Herb. Wagensommer*), and A. Soldano (*Herb. Soldano*), were physically (about 95% of the samples) examined and studied (Figure 1).

We included in the morphological study 195 herbarium specimens (56 of *T. striatus* subsp. *striatus* [STR], 44 of *T. striatus* subsp. *acicularis* [ACI], 49 of *T. spinulosus* [SPI], and 46 of *Thymus* from the Murge area [APU]), from 43 populations. The populations studied were selected to include the type localities (or adjacent areas) and to cover the whole distribution range of the four taxa included in this study.

The co-existence of hermaphrodite and female individuals (gynodioecy) in natural populations is very common in *Thymus* [47,48,49,50]. The female individuals (with reduced anthers and little or no viable pollen) have a smaller calyx and corolla than hermaphrodite ones. Therefore, for morphological analyses, we selected only hermaphrodite specimens.

### 2.2. Morphometric Analyses

**Figure 2:**
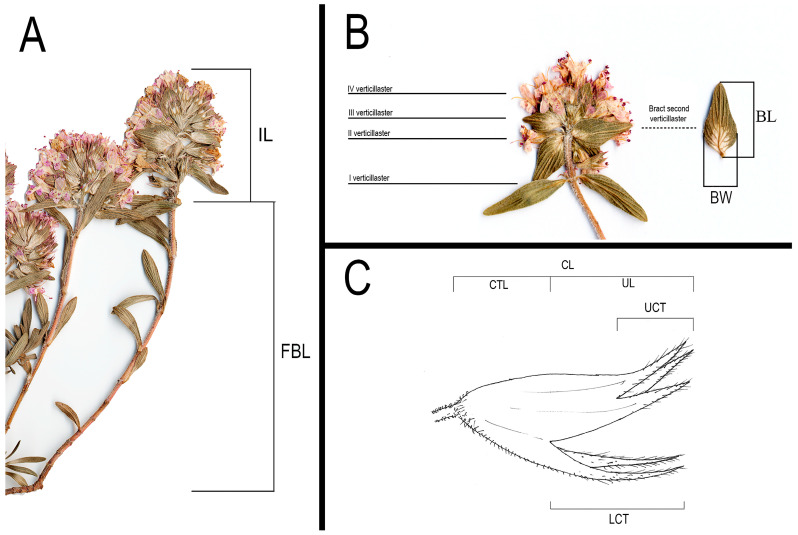
Quantitative characters measured in this study (the *taxon* referenced in the table is *T. striatus* subsp. *striatus*): (**A**) “IL”, inflorescence length; “FBL”, flowering branch length. (**B**, modified by [8]) “BL”, bract length; “BW”, bract width. (**C**, modified by [8]) “CL”, calyx length; “CTL”, calyx tube length; “UCT”, upper calyx teeth length; “LCT”, lower calyx teeth length; “UL”, upper limb length. Illustration of the calyx (**C**) by Fabrizio Bartolucci.

In total, 21 qualitative and quantitative morphological characters and 5 calculated ratios (Table 1) were studied, with a resulting dataset of 195 cases × 26 variables. The characters were selected according to their common use to discriminate *taxa* within the genus *Thymus* [10,11,12,21,23,27,45,51]. The micromorphological characters were examined under a stereomicroscope, and parameters were measured with a grid 12 mm/120 or simply with a ruler. Some of the measured quantitative characters of inflorescence, flowering branch, calyx, and bracts (floral leaves) are represented in Figure 2. The measurements of the bracts refer to those of the second verticillaster of the inflorescence.

The dataset was subjected to a series of refinements to prepare it for multivariate analyses. The first step was to remove cases with missing data in measured traits, obtaining a dataset of 190 cases (56 of *T. striatus* subsp. *striatus* [STR], 39 of *T. striatus* subsp. *acicularis* [ACI], 49 of *T. spinulosus* [SPI], 46 of *Thymus* from Murge area [APU]) × 26 variables. Then, for all quantitative characters, the normality of distribution within groups was tested with the Shapiro–Wilk test. Then, univariate analysis was used to evaluate correlated variables or variables showing non-significant differences among the studied groups. The quantitative variables were tested with the Kruskal–Wallis test followed by Dunn’ test with a Bonferroni-corrected post hoc pairwise comparison, and the qualitative variables were subjected to the chi-squared test. Finally, we performed a correlation test via Spearman’s non-parametric coefficient, which aimed to exclude characters with a correlation score > 0.8. The above-mentioned statistical analyses were carried out using SPSS Statistics for Data Analysis v.25 [52].

The resulting dataset was subjected to principal coordinate analysis (PCoA) to visualize the patterns of morphological variation among the studied groups. The similarity matrix was calculated using the Gower coefficient, which is suitable for mixed data [53,54]. Linear Discriminant Analysis (LDA) was performed, taking into account the morphological groups individuated with the PCoA. Multivariate analyses were carried out using PAST package v5.2.1 [55].

## 3. Results

### Morphometric Analyses

According to the Kruskal–Wallis and chi-squared tests, all the studied morphological characters had statistically significant differences among the compared groups. The correlation test indicated that the calyx length (CL) was highly correlated to upper limb length (UL) (r = 0.890; *p* < 0.01). Therefore, the upper limb length (UL) was excluded from subsequent analyses. After filtering, we obtained a data matrix comprising 190 cases × 25 morphological characters (15 quantitative, 5 qualitative, and 5 ratios).

**Figure 3:**
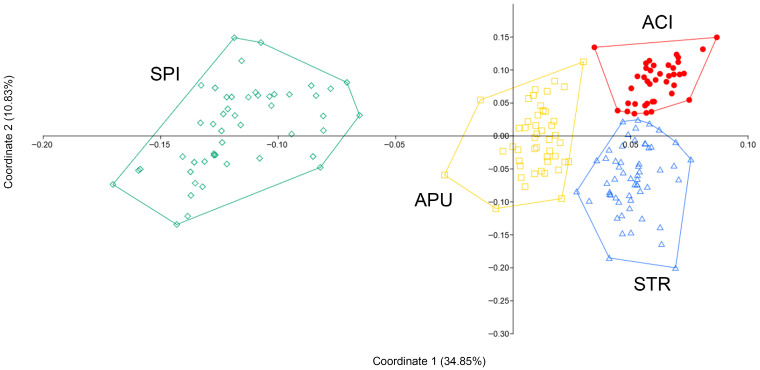
Biplot for the PCoA performed with Gower’s distance for mixed data. *Thymus spinulosus* [SPI] (green diamonds), *Thymus* from the Murge area [APU] (yellow squares); *T. striatus* subsp. *acicularis* [ACI] (red dots), *T. striatus* subsp. *striatus* [STR] (blue triangles).

The first two axes of the PCoA explained 45,68% of the total variation of the dataset. The scatterplot shows, on the first two axes, a clear separation of the four *taxa* (SPI, APU, STR and ACI). No overlapping areas among individuals were found (Figure 3).

The LDA based on the 15 quantitative characters and 5 ratios performed on the four groups individuated by the PCoA resulted in an 88.42% correct classification rate using the jackknifed classification method. *Thymus striatus* subsp. *acicularis* (ACI) was the best-supported group, with 100% of cases correctly classified (39 out of 39 cases), followed by *Tymus* from the Murge area (APU), with 86.9% correct classification (40 out of 46 cases); *T. striatus* subsp. *striatus* (STR), with 85.71% correct classification (48 out of 56 cases); and *T. spinulosus* (SPI), with 83,67% correct classification (41 out of 49 cases) (Table 2).

Based on the Kruskal–Wallis and Dunn’s tests with Bonferroni correction, we conclude that the mean ranks of FBL are significantly different (*p* < 0.05) between ACI-STR; LW, LL, FL, and CTL are significantly different between ACI-SPI, ACI-STR, and ACI-APU; IL and VN are significantly different between ACI-APU, ACI-SPI, ACI-STR, APU-SPI, and STR-SPI; PL and LCT are significantly different between ACI-APU, ACI-SPI, ACI-STR, STR-APU, and STR-SPI; BPL and FN are significantly different between ACI-SPI, ACI-STR, APU-SPI, and APU-STR; CL and UL are significantly different between ACI-SPI, ACI-STR, ACI-APU, and SPI-STR; UCT is significantly different between SPI-STR, ACI-STR, and APU-STR; BL is significantly different between ACI-SPI, ACI-STR, APU-SPI, APU-STR, and STR-SPI; BW is significantly different between SPI-STR, APU-STR, and ACI-STR; LL/LW is significantly different between STR-ACI, APU-ACI, and SPI-ACI; CTL/CL is significantly different between APU-STR, APU-SPI, ACI-SPI, and STR-SPI; LCT/UL is significantly different between SPI-STR and APU-STR; BW/LW is significantly different between SPI-STR, SPI-ACI, APU-STR, and APU-ACI; BL/BW is significantly different between STR-APU, STR-SPI, ACI-SPI, and APU-SPI. Descriptive statistics for each morphometric character in the four taxa studied are presented in Table 3.

## 4. Taxonomic Treatment

**Figure 4:**
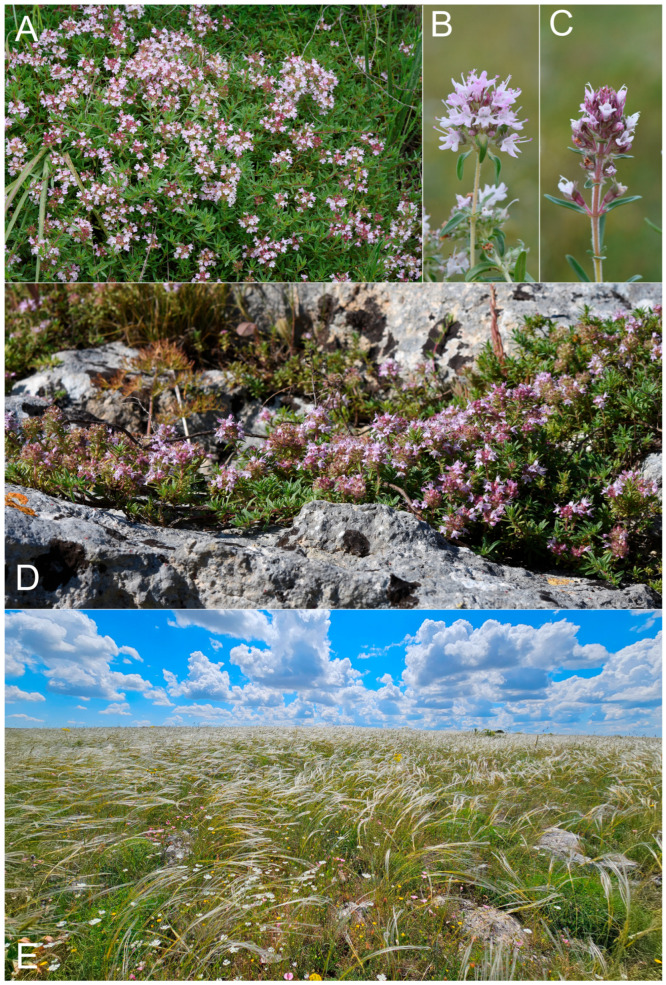
Habitat and flowering plants of *Thymus apulus*: (**A**) *T. apulus* growing in xeric grasslands of type locality (southern Italy, Apulia, Murge di Parisi Vecchio in loc. Cento Tomoli); (**B**) close-up of hermaphroditic plant of *T. apulus* (southern Italy, Apulia, Murge di Parisi Vecchio in loc. Cento Tomoli); (**C**) close-up of female plant of *T. apulus* (southern Italy, Apulia, Murge di Parisi Vecchio in loc. Cento Tomoli); (**D**) *T. apulus* growing in rocky outcrops at Murge di Matera (southern Italy, Basilicata, Matera); (**E**) preferential habitat of *T. apulus* referring to sub-Mediterranean xeric grasslands dominated by *Stipa austroitalica* (southern Italy, Apulia, Alta Murgia, Podere San Michele). Photos by F. Bartolucci.

***Thymus apulus*** Bartolucci & F.Conti, *sp. nov.* (Figure 4)

Type: Italy. Apulia, Altamura (Bari), Murge di Parisi Vecchio in loc. Cento Tomoli (WGS84 40.8904° N, 16.45328° E), praterie a *Stipa austroitalica*, 547 m, 13 May 2025, *F. Bartolucci s.n.* (holotype: APP No. 73588, see Supplementary Material File S2; isotypes: APP Nos. 73589, 73590, 73591, and FI090834).

**Figure 5:**
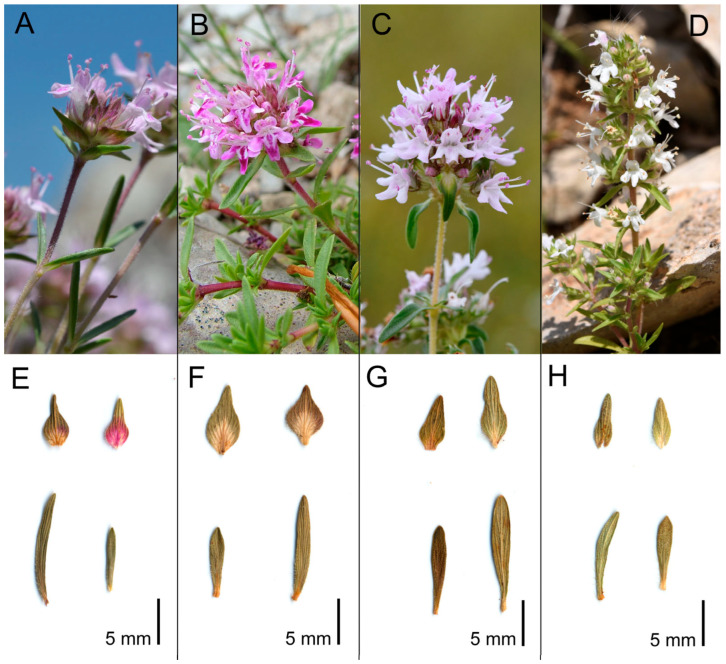
General view of bracts and leaves of the species studied here. (**A**,**E**) *Thymus striatus* subsp. *acicularis* (central Italy, Abruzzo, Monte della Selva, Barisciano, L’Aquila); (**B**,**F**) *T. striatus* subsp. *striatus* (*locus classicus*, southern Italy, Campania, Monte Faito di Castellammare, Napoli); (**C**,**G**) *T. apulus* (*locus classicus*, southern Italy, Apulia, Murge di Parisi Vecchio, Altamura, Bari); (**D**,**H**) *T. spinulosus* (*locus classicus*, southern Italy, Apulia, Monte Tre Titoli, Accadia, Foggia). Photos by *F. Bartolucci*.

Diagnosis: *Thymus apulus* is distinguished from *T. spinulosus* by its pink corolla (vs. white), leaves, calyx, and corolla with sparse oil glands (vs. dense oil glands), calyx greenish tinged with purple (vs. greenish), inflorescence capituliform to elongated (vs. usually interrupted), shorter bracts, and smaller calyx tube length/calyx length and bract length/bract width ratios; it is distinguished from *T. striatus* subsp. *striatus* by its pink corolla (vs. pink to purplish), leaves with non-parallel lateral veins (vs. parallel), smaller lower and upper calyx teeth, shorter and narrower bracts, smaller bract width/middle cauline leaf width ratio and higher bract length/bract width ratio; it is distinguished from *T. striatus* subsp. *acicularis* by its pink corolla (vs. pink to purplish), leaves with non-parallel lateral veins (vs. parallel), longer inflorescence, calyx and corolla, and smaller ratio bract width/middle cauline leaf width (Figure 5, Table 3).

Description: Loosely mat-forming, with woody procumbent or prostrate shoots. Flowering stems (20)32–55(120) mm, sometimes with axillary leaf-clusters at least at the base, pubescent all around. Leaves, elliptic-oblanceolate, sub-sessile, herbaceous, glabrous on both sides or sparsely hirsute above, ciliate at base, with a prominent midvein and less distinct non-parallel lateral veins (better visible on dried specimens), sparse (rare) sessile oil glands; middle cauline leaves (5.8)8–11.3(16) × (1.3)1.60–2(2.5) mm, (3.4)4.8–5.9(8) times longer than wide. Inflorescence capituliform to elongated (rarely interrupted) (9)12–16(54) mm long, verticillasters 10–60 flowers; bracts (second verticillaster) narrowly to widely ovate (3.8)5–6(12) × (1.5)2–2.6(3.5) mm, different to the cauline leaves, rare ± similar (only the basal ones), ciliate at lower half, with a conspicuous midvein and veinlets, with sparse oil dots, glabrous to sparsely hirsute. Bracteoles linear to linear-lanceolate, (0.6)1–1.3(2) mm long, usually green, shorter than pedicels, ciliate. Pedicel (1)1.8–2.3(4) mm long. Calyx bilabiate, (3.8)4.4–5(6) mm long, cylindrical, hairy all around, greenish tinged with purple; calyx tube (1.1)1.6–1.9(2.5) mm long, upper calyx teeth ciliate (0.7)1–1.2(1.8) mm long, lower ones ciliate (1.6)2.2–2.6(3.4) mm long, upper limb (2.2)2.7–3(4.1) mm long. Corolla bilabiate, (5)5.5–6.2(7) mm long, pink, pubescent, exceeding calyx. Nutlets elliptic-ovoid, (0.6)0.75–0.9(1) mm, blackish.

Etymology: *Thymus apulus* is named after “Apulia”, a territory inhabited by the Apuli, an ancient Italic tribe, currently known with the Italian name Puglia, an administrative region located in the southern peninsular Italy.

Habitat: Xeric grasslands and rocky outcrops; altitude ranges from 350 to 650 m a.s.l. The preferential habitat is characterized by sub-Mediterranean xeric grasslands dominated by *Stipa austroitalica* Martinovský, corresponding to *Hippocrepido glaucae*-*Stipion austroitalicae* Forte & Terzi 2005 alliance, *Acino suaveolentis*-*Stipetum austroitalicae* Forte & Terzi 2005, and *Chamaecytiso spinescentis-Stipetum austroitalicae* Terzi & Forte 2005 associations [37,43]. In rocky outcrops, it grow in garrigues corresponding to *Cytiso spinescentis-Saturejion montanae* Pirone & Tammaro 1997 alliance and *Rhamno saxatilis-Saturejetum montanae* Tomaselli, Silletti & Forte 2021 association [39,56].

Phenology: Flowering from late April to May, fruiting from late May to June.

**Figure 6:**
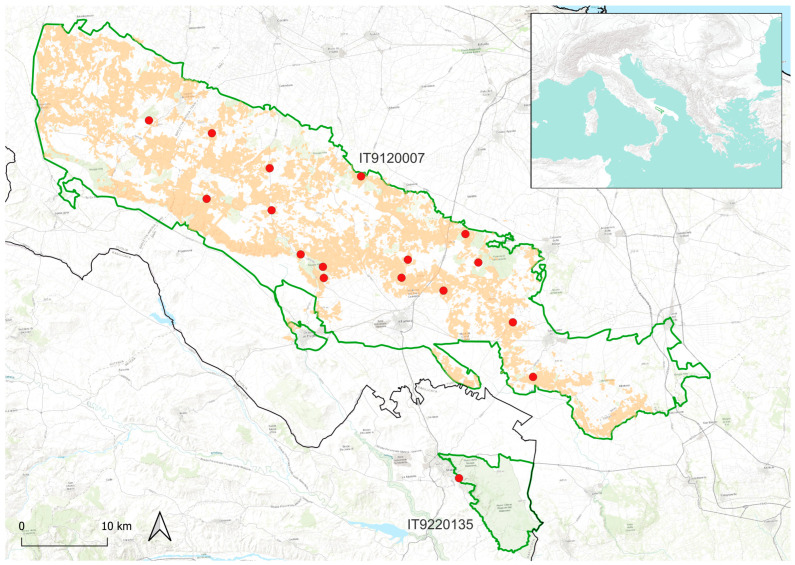
Distribution map of *Thymus apulus* based on the herbarium specimens studied (red dots). The boundaries of the NATURA 2000 network are highlighted in green (SCI/SPA IT9120007 “Alta Murgia” and SCI/SPA IT9220135 “Gravine di Matera”). The distribution of Habitat 62A0 “Eastern sub-Mediterranean dry grasslands (*Scorzoneratalia villosae*)” within the NATURA 2000 network (SCI/SPA IT9120007 “Alta Murgia” and SCI/SPA IT9220135 “Gravine di Matera”) is reported in orange [61].

Distribution: Endemic to Murge hill area (southern Italy) within the Alta Murgia National Park (Apulia) and Murgia Materana Park (Basilicata) (Figure 6).

Conservation status: All known localities of *T. apulus* are included in the NATURA 2000 network (SCI/SPA IT9120007 “Alta Murgia” and SCI/SPA IT9220135 “Gravine di Matera”). *Thymus apulus* grows preferentially in plant communities dominated by *Stipa austroitalica* belonging to Habitat 62A0, “Eastern sub-Mediterranean dry grasslands (*Scorzoneratalia villosae*)”, included in Annex I of the Habitats Directive (92/43/EEC).

The Extent of Occurrence (EOO) of *T. apulus* is 730 km^2^, and the area of occupancy (AOO) is 64 km^2^ (16 cells grid 2 × 2 km), calculated with GeoCAT (Geospatial Conservation Assessment Tool) software [57]. Despite the reduced EOO and AOO, the population demonstrates demographic stability. No evidence of ongoing decline has been recorded, and no discernible anthropogenic or natural pressures and threats have been identified at present.

The species’ ecology is closely associated with Habitat 62A0, which, according to the latest national report submitted to the European Commission about the habitats of Community interest, showed a favorable conservation status and an increasing trend within the Mediterranean biogeographical region during the 2013–2018 reporting period [58]. Nevertheless, this habitat represents an ecologically sensitive system that persists due to a finely tuned equilibrium maintained by a complex interplay of enduring natural and anthropogenic influences [59,60].

Distribution data for Habitat 62A0 within the Natura 2000 network (Figure 6) [61] suggest that *T. apulus* may be more widespread and abundant than current records indicate. Therefore, according to IUCN criteria [62], the species could be assessed as Least Concern (LC) at the global level.

*Key to taxa of Thymus sect. Hyphodromi distributed in Italy*

(Note: measurements refer to hermaphroditic plants only.)

1. Plants densely covered with reddish or pale sessile oil glands, leaves sparsely hairy above, inflorescence usually interrupted, calyx greenish, corolla white. ***T. spinulosus.***

1. Plants with rare, pale sessile oil glands, leaves glabrous or sparsely hairy, inflorescence capituliform to elongated (rarely interrupted), calyx greenish tinged with purple or purple, corolla pink to purplish.…………………………………..…….….………..………....2.

2. Leaves elliptic-spathulate to oblanceolate, glabrous or hairy, with non-parallel lateral veins, herbaceous to coriaceous and ± fleshy; corolla usually pink ………….………..3.

2. Leaves linear, falcate to subspathulate, glabrous, with parallel lateral veins, coriaceous but not fleshy; corolla pink to purplish……………………………………………..…..4.

3. Leaves elliptic-spathulate, petiolate, coriaceous, and ± fleshy; inflorescence capituliform (7)10–12(30) mm long; flowering branches (14)22–35(50) mm long; middle cauline leaves (5)6.7–7.9(10.5) × (1.3)1.9–2.3(3) mm, (2.5)3.05–4(4.69) longer than wide; calyx (4.6)5–5.5(6) mm long, lower calyx teeth (2.5)2.9–3.1(3.8) mm long, upper limb (2.9)3.1–3.4(4) mm long; corolla (5.5)6.5–7.5(8.5) mm long (endemic to Sicily)...... ***T. paronychioides.***

3. Leaves elliptic-oblanceolate, sub-sessile, herbaceous; inflorescence capituliform to elongated (rarely interrupted) (9)12–16(54) mm long; flowering branches (20)32–55(120) mm long; middle cauline leaves (5.8)8–11.3(16) × (1.3)1.60–2(2.5) mm, (3.4)4.8–5.9(8) longer than wide; calyx (3.8)4.4–5(6) mm long, lower calyx teeth (1.6)2.2–2.6(3.4) mm long, upper limb (2.2)2.7–3(4.1) mm long; corolla (5)5.5–6.2(7) mm long (endemic to Apulia and Basilicata)…………………………………………………………………………….….***T. apulus.***

4. Plants with robust habit, leaves linear to subspathulate (1.1)1.7–2 (3.1) mm wide; bracts (2)3.4–4(5) mm wide; calyx (3.8)4.6–5.1(6) mm long, lower calyx teeth (2.1)2.40–2.80(3.5) mm long, upper calyx teeth (1.1)1.3–1.5(2) mm long; corolla (3.80)4.6–5.1(6) mm long ……………………………………………………………..…....***T. striatus*** subsp. ***striatus.***

4. Plants with slender habit, leaves linear, ± falcate (0.7)1–1.3(1.6) mm wide; bracts (1.5)2–2.65(3.6) mm wide; calyx (3)3.6–4.1(4.5) mm long, lower calyx teeth (1.7)1.95–2.1(2.5) mm long, upper calyx teeth (0.8)1–1.25(1.7) mm long; corolla (3)3.60–4.1(45) mm long …………………………………………………...……………***T. striatus*** subsp. ***acicularis.***

## 5. Discussion

Morphometric analyses provide evidence that the *Thymus* populations from the Murge hill area (Apulia and Basilicata, southern Italy) should be recognized as a new species, here named *T. apulus*, strictly endemic to Alta Murgia National Park and Murgia Materana Park. All known localities of *Thymus apulus* are included within the Natura 2000 network (SCI/SPA IT9120007 “Alta Murgia” and SCI/SPA IT9220135 “Gravine di Matera”).

*Thymus apulus* grows preferentially in species-rich endemic communities dominated by *Stipa austroitalica* corresponding to Habitat 62A0, “Eastern sub-Mediterranean dry grasslands (*Scorzoneratalia villosae*)”, included in Annex I of the Habitats Directive (92/43/EEC). These dry grassland communities show a rich contingent of Italian rare and endangered endemic species that significantly contribute to the conservation value of this habitat [7,37,43,63,64]: *Carduus nutans* subsp. *perspinosus* (Fiori) Arènes, *Centaurea brulla* Greuter, *Erysimum crassistylum* subsp. *garganicum* Peccenini & Polatschek, *Euphorbia japygica* Ten. subsp. *japygica*, *Gelasia villosa* subsp. *columnae* (Guss.) Bartolucci, Galasso & F.Conti, *Iris pseudopumila* Tineo, *Picris scaberrima* Guss., *Rhaponticoides centaurium* (L.) M.V.Agab. & Greuter, *Stipa austroitalica* Martinovský, and *Thymus spinulosus*. In this habitat or in garrigues in rocky areas where *T. apulus* is found, there are also many species of significant phytogeographical interest, some of which are exclusive to southern Italy, such as *Asyneuma limonifolium* (L.) Janch. subsp. *limonifolium*, *Euphorbia apios* L., *Helianthemum jonium* Lacaita & Grosser, and *Ziziphora suaveolens* (Sm.) Melnikov.

*Thymus apulus* has morphological characters, such as leaves with a prominent midvein and usually weak, non-parallel lateral veins, bracts different from the leaves, that place it, according to Jalas’s classification [30], within the *T*. subsect. *Serpyllastrum* [20,30]. Actually, also *T. spinulosus*, which is traditionally placed in the *T.* subsect. *Subbracteati* [20,21], appears to be more appropriately classified within the *T*. subsect. *Serpyllastrum*. The supraspecific classification of *Thymus* is based exclusively on qualitative morphological traits and undoubtedly requires revision through an integrative taxonomic framework incorporating molecular analyses.

*Thymus apulus* is distinguished from *T. spinulosus* by its pink corolla (vs. white), leaves, calyx and corolla with sparse oil glands (vs. dense oil glands), capituliform to elongated inflorescence, shorter bracts, greenish calyx tinged purple (vs. greenish), and smaller calyx tube length/calyx length and bract length/bract width ratios. A noteworthy feature is the sympatric occurrence of *T. apulus* and *T. spinulosus*. Within the surveyed Murge area, these two species typically coexist in sub-Mediterranean xeric grasslands dominated by *Stipa austroitalica*. Although hybridization is a common phenomenon among sympatric *Thymus* species, morphologically intermediate individuals, which could represent potential hybrids, are rare. This phenomenon is attributed to the variation in flowering phenology between the species, differing by up to approximately one month. For instance, during early May, hermaphroditic individuals of *T. apulus* reach full anthesis (with female ones starting to flower several days before hermaphrodites), whereas individuals of *T. spinulosus* remain non-flowering. Asynchronous anthesis periods function as a reproductive isolating mechanism, preserving distinct species boundaries despite sympatry, thereby reinforcing their separate taxonomic identities within shared ecological niches. This explains why, aside from potential misidentifications, in phytosociological surveys conducted in the Murge area between mid-May and June, almost exclusively *T. spinulosus* has been reported [36,37,39,41].

Morphometric analyses also revealed significant differences between *T. apulus* and *T. striatus*, which also exhibit distinct ecological preferences. *Thymus striatus* is a mountain orophyte, typically inhabiting calcareous and serpentine rocky and stony slopes within an altitudinal range from (300) 500 to 1500 (2300) m a.s.l. *Thymus apulus* is distinguished from *T. striatus* by its pink corolla (vs. pink to purplish) and leaves with non-parallel lateral veins (vs. parallel), narrower bracts, smaller bract width/middle cauline leaf width ratio and higher bract length/bract width ratio.

**Figure 7:**
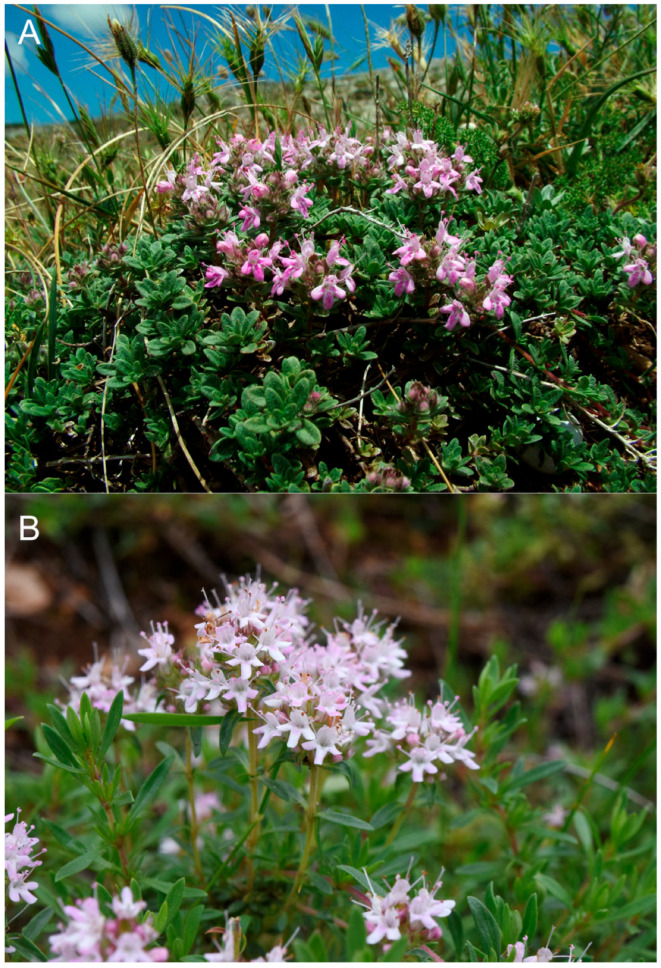
(**A**) *Thymus paronychioides* (*locus classicus*, southern Italy, Sicily, Monte Busambra, Palermo); (**B**) *T. apulus* (southern Italy, Apulia, Alta Murgia al Bosco “Trullo di Sotto”, Gravina in Puglia, Bari). Photos by F. Bartolucci.

*Thymus apulus* also differs from *T. paronychioides* (Figure 7), an endemic species to central-northern Sicily, which grows on dry rocky slopes at altitudes from 600 to 1800 m [21,22]. *Thymus paronychioides* is mainly distinguished by its prostrate habit, leaves elliptic-spathulate, petiolate, coriaceous, and ± fleshy with prominent lateral veins, shorter flowering stems (sometimes amphitrichous on the lower internodes), and generally larger calyx and corolla.

An interesting ecological similarity can be observed with the geographical vicariant *T. aznavourii*, a Balkan endemic species (Eastern Thrace) [23,33], which inhabits plant communities similar to those of *T. apulus*, attributable to Habitat 62A0, “Eastern sub-Mediterranean dry grasslands (*Scorzoneratalia villosae*)”. Despite their ecological affinity, *T. aznavourii* differs distinctly in morphological terms from *T. apulus*, mainly due to its white corolla, pale greenish calyx never tinged with purple, and upper teeth 0.5–0.8 mm long that are not ciliate [33].

## 6. Conclusions

The present study demonstrates that the *Thymus* populations from the Murge hill area are sufficiently different from other known *Thymus* species belonging to *T.* sect. *Hyphodromi* occurring in Italy, supporting the description of *T. apulus* as a new species to science. This study also provides basic information about the distribution and ecology of the new species, which are essential for setting conservation priorities. Furthermore, the conservation status assessment of *T. apulus* according to IUCN categories and criteria are proposed and discussed. Additionally, we provide an identification key for dried herbarium specimens of *Thymus taxa* belonging to *T.* sect. *Hyphodromi* in Italy. This helps in the correct identification of these *taxa*, which is essential for monitoring and conservation efforts.

## Figures and Tables

**Table 1:** List of morphological characteristics measured: characters code; description of the characters, and variable type.

Characters Code	Description of the Characters [Unit of Measure or Characters States]	Type
CC	Corolla color: 0 = white; 1 = pink to purplish	Qualitative
EH	Eglandular trichomes on the adaxial, middle cauline leaf, surface: 0 = absent; 1 = present	Qualitative
OD	Sessile oil glands (leaves, calyx, and corolla): 0 = rare; 1 = abundant	Qualitative
LV	Lateral veins of leaves (best seen in dried specimens): 0 = parallel; 1 = non-parallel	Qualitative
IS	Inflorescence shape: 0 = capituliform to elongated (basal verticillaster remote); 1 = interrupted (2–3 verticillasters remote below oblong terminal head)	Qualitative
FBL	Flowering branches length (without inflorescence) (mm)	Quantitative continuous
LW	Middle cauline leaf width (mm)	Quantitative continuous
LL	Middle cauline leaf length (mm)	Quantitative continuous
IL	Inflorescence length (mm)	Quantitative continuous
FL	Corolla length (mm)	Quantitative continuous
PL	Pedicel length (mm)	Quantitative continuous
BPL	Bracteole of the pedicel length (mm)	Quantitative continuous
CL	Calyx length (mm)	Quantitative continuous
CTL	Calyx tube length (mm)	Quantitative continuous
UCT	Upper calyx teeth length (mm)	Quantitative continuous
LCT	Lower calyx teeth length (mm)	Quantitative continuous
UL	Upper limb length (mm)	Quantitative continuous
BL	Bract length (second verticillaster of the inflorescence) (mm)	Quantitative continuous
BW	Bract width (second verticillaster of the inflorescence) (mm)	Quantitative continuous
FN	Number of flowers (single inflorescence)	Quantitative discrete
VN	Number of verticillasters (single inflorescence)	Quantitative discrete
LL/LW	Middle cauline leaf length/Middle cauline leaf width	Ratio
CTL/CL	Calyx tube length/calyx length	Ratio
LCT/UL	Lower calyx teeth length/upper limb length	Ratio
BW/LW	Bract width/Middle cauline leaf width	Ratio
BL/BW	Bract length/bract width	Ratio

**Table 2:** Jackknifed confusion matrix of the LDA: *Thymus spinulosus* [SPI], *Thymus* from the Murge area [APU], *T. striatus* subsp. *acicularis* [ACI], and *T. striatus* subsp. *striatus* [STR]. Rows show the membership of each group individuated by PCoA, whereas columns show the membership predicted by the classification model.

	ACI	APU	SPI	STR	Total
ACI	39	0	0	0	39
APU	0	40	4	2	46
SPI	2	4	41	2	49
STR	3	3	2	48	56
Total	44	47	47	52	190

**Table 3:** Comparisons of each morphological character among the four *Thymus* taxa involved in this study. For quantitative continuous characters, mean ± standard deviation are reported. For quantitative integer characters, *minimum* and *maximum* values are reported.

Character Code	*Thymus* from Murge Area (APU)	*T. spinulosus* (SPI)	*T. striatus* subsp. *striatus* (STR)	*T. acicularis* subsp. *acicularis* (ACI)
CC	pink	white	pink to purplish	pink to purplish
EH	absent or present	present	absent	absent
OD	rare	abundant	rare	rare
LV	non-parallel	non-parallel	parallel	parallel
IS	capituliform to elongated (rarely interrupted)	elongated to interrupted	capituliform (rarely elongated)	capituliform (rarely elongated)
FBL	46.80 ± 19.15	46 ± 21.82	51.93 ± 18.33	36.00 ± 15.5
LW	1.77 ± 0.28	1.76 ± 0.38	1.89 ± 0.36	1.14 ± 0.21
LL	9.57 ± 1.92	10.1 ± 1.92	9.99 ± 1.76	8.29 ± 1.98
IL	15.58 ± 7.97	26.18 ± 13.35	15.82 ± 6.28	8.23 ± 3.34
FL	5.91 ± 0.58	5.61 ± 0.48	5.75 ± 0.46	4.83 ± 0.54
PL	2.04 ± 0.55	2.11 ± 0.64	1.61 ± 0.48	1.26 ± 0.36
BPL	1.17 ± 0.33	1.4 ± 0.37	1.41 ± 0.40	1.05 ± 0.35
CL	4.71 ± 0.45	4.55 ± 0.42	4.86 ± 0.46	3.87 ± 0.33
CTL	1.80 ± 0.26	1.81 ± 0.24	1.88 ± 0.24	1.44 ± 0.18
UCT	1.14 ± 0.23	1.09 ± 0.15	1.44 ± 0.21	1.13 ± 0.21
LCT	2.37 ± 0.39	2.21 ± 0.24	2.58 ± 0.31	2.02 ± 0.20
UL	2.89 ± 0.33	2.76 ± 0.31	2.97 ± 0.30	2.44 ± 0.29
BL	5.57 ± 1.04	6.44 ± 0.89	7.44 ± 1.45	5.39 ± 0.76
BW	2.32 ± 0.43	2.22 ± 0.38	3.67 ± 0.59	2.43 ± 0.43
VN	3–7	4–9	3–6	2–4
FN	10–60	20–70	16–68	8–36
LL/LW	5.45 ± 0.92	5.94 ± 1.32	5.37 ± 0.95	7.5 ± 2.29
CTL/CL	0.31 ± 0.08	0.49 ± 0.15	0.39 ± 0.03	0.37 ± 0.04
LCT/UL	0.82 ± 0.10	0.81 ± 0.09	0.87 ± 0.08	0.84 ± 0.09
BW/LW	1.33 ± 0.27	1.31 ± 0.32	2.00 ± 0.45	2.19 ± 0.47
BL/BW	2.45 ± 0.57	2.97 ± 0.63	2.07 ± 0.51	2.26 ± 0.41

## Data Availability

The data presented in the current study are available within the article and Supplementary Materials.

## Supplementary Materials

The following supporting information can be downloaded at https://www.mdpi.com/article/10.3390/plants14233584/s1, File S1: List of the herbarium specimens examined; File S2: Image of the holotype kept at APP herbarium.
