# Supplementary material for: Thymus apulus (T. sect. Hyphodromi, Lamiaceae), a New Species from Southern Italy
Source: Plants (Basel). 2025 Nov 24;14(23):3584. doi: 10.3390/plants14233584 (PMC12693766; doi:10.3390/plants14233584)
Supplement: Supplementary file 1 [file plants-14-03584-s001.zip › plants-4001191 supplementary/Sup_fileS1.pdf]

**Article:** *Thymus apulus (T. sect. Hyphodromi, Lamiaceae), a New Species from Southern Italy*

**Authors:** Fabrizio Bartolucci & Fabio Conti

**Supplementary File.**

**List of the specimens examined. The herbarium specimens used for morphometric analyses are marked with an asterisk "*".**

***Thymus apulus*** Bartolucci & F.Conti

**ITALY: Apulia.** il Pulo di Altamura (Altamura, Bari), rupi e pascoli sassosi, 475 m, 07/V/2009, *F. Bartolucci, F. Conti, M. Iocchi* (APP, 4 sheets)*; loc. Murge di Parisi Vecchio, (Altamura, Bari), rupi e pascoli sassosi, 509 m, 07/V/2009, *F. Bartolucci, F. Conti, M. Iocchi* (APP, 19 sheets)*; pascoli alla Murgia di Cassano (Terra di Bari), 7/VI/1896, *Palanza* (FI sub *T. striatus*); Murgia di S. Elia, 16/VI/1950, *F. Scaramozzi* (BI No. 26368 sub *T. striatus* var. *spinulosus*); Toritto, 03/V, *s. coll.* (BI No. 8934 sub *T. striatus* var. *spinulosus*); presso Toritto, 17/V/1940, *s. coll.* (BI No. 8936); Murge di Altamura, Bari, 17/V/1943, *s. coll.* (BI No. 8933 sub *T. striatus* var. *spinulosus*); Cassano, bosco Santoro, 13/V/1950, *V. Armeniso* (BI Nos 18447, 18448 sub *T. striatus* var. *spinulosus*); Pulicchio, Gravina in Puglia (Bari) (UTM ED50 4529357, 620072), prateria arida, 18/IV/2010, *F. Mantino* (BI No. 35987 sub *T. spinulosus*); Azienda Donnapaola (Grumo Appula, Bari) (40°54′50′′, 16°39′20′′), prateria, 21/V/2022, *G. Pazienza* (BI Nos 58402, 58394, 58395 sub *T.* sect. *Hyphodromi*); Lamalunga (Santeramo in Colle, Bari), prateria a *Stipa austroitalica* e *Scorzonera* sp.pl., 465 m, 12/V/2018, *F. Bartolucci et al.* (APP Nos 60992-61011; PI, *Herb. Cancellieri*, IT)*; C.da Pantano, 12/V/2008, *R. Di Pietro* (HFLA, 4 sheets, sub *T.* cfr. *spinulosus*); Altamura (Bari), presso Masseria Chinunno (WGS84 40.88067°N, 16.66914°E), prateria a *Stipa austroitalica,* 462 m, 14/V/2025, *F. Bartolucci* (APP, 2 sheets)*; Altamura (Bari), loc. Scalcione (WGS84 40.8548°N, 16.61715°E), stipeto a *Stipa austroitalica*, 405 m, 13/V/2025, *F. Bartolucci* (APP); Altamura (Bari), presso Masseria Caputi (WGS84 40.9552°N, 16.38874°E), stipeto a *Stipa austroitalica*, 605 m, 12/V/2025, *F. Bartolucci* (APP); Gravina in Puglia (Bari), loc. Pulicchio (WGS84 40.9057°N, 16.42426°E), stipeto a *Stipa austroitalica*, 552 m, 12/V/2025,

*F. Bartolucci* (APP, 3 sheets)*; Altamura (Bari), Murge di Parisi Vecchio in loc. Cento Tomoli (WGS84 40.8904°N, 16.45328°E), praterie a *Stipa austroitalica*, 547 m, 13/V/2025, *F. Bartolucci* (APP nos 73588, 73589, 73590, 73591)*; Andria (Bari), presso Piano di Citulo (WGS84 40.0589°N, 16.22934°E), stipeto a *Stipa austroitalica*, 518 m, 12/V/2025, *F. Bartolucci* (APP, 18 sheets)*; Santeramo in Colle (Bari), presso Iazzo Torretta (WGS84 40.7555°N, 16.73049°E), stipeto a *Stipa austroitalica*, 453 m, 14/V/2025, *F. Bartolucci* (APP); Bitonto (Bari), Murgia del Ceraso (WGS84 40.9823°N, 16.51607°E), 407 m, 14/V/2025, *F. Bartolucci* (APP, 3 sheets)*; Gravina in Puglia (Bari), presso Bosco Trullo di Sotto (WGS84 40.972387°N, 16.300733°E), rupi, 588 m, 15/V/2025, *F. Bartolucci* (APP, 6 sheets)* **Basilicata**. Matera, Gravina di Matera (WGS84 40.6556°N, 16.61730°E), stipeto a *Stipa austroitalica* e rupi, 395 m, 13/V/2025, *F. Bartolucci* (APP, 4 sheets)*;

***Thymus spinulosus*** Ten.
**ITALY: Lazio.** Faete, Monte Nero e Monte Coccia (Arpino), V-VI/1896, *G. Falqui* (PORUN); **Campania**. Montevergine presso Avellino, 28/VI/1897, *M. Guadagno* (PI); Lacedonia (Avellino), pascoli aridi, 850 m, 26.VI. 2014, *M. Terzi, V. Tomaselli* (FI); **Apulia**. nei pressi di Masseria Montanaro (Mottola, Taranto) (ED50 674694E 4504150N), prateria da alta rocciosità, 13/V/2010, *F. Carruggio, F. Mantino* (BI); loc. Chiancata Avatra (San Giovanni Rotondo, Foggia) (ED50 566044E 4613782N), gariga ad elevata rocciosità, 31/V/2008, *F. Mantino* (BI); a monte di Masseria Bramante (San Giovanni Rotondo, Foggia) (ED50 567269E 4612488N), prateria arida, 14/IV/2007, *F. Mantino* (BI); Patemisco, 30/III/1949, *s.coll.* (BI); Gravina della Madonna della Scala (Massafra), 28/V/1953, *V. Grimaldi* (BI); Ruvo, Parco del Conte, 18/V/1949, […] (BI); Segezia, pascoli dell'Ovile Nazionale, 30/IV/1948, *Sarfatti* (BI); *ibidem*, 13/V/1949, *Sarfatti* (BI); Masseria Riccardo […], 29/V/1953, […] (BI); sulle […] di Patemisco, 17/V/1948, *s.coll.* (BI); Cassano, bosco Santoro, 13/V/1950, *V. Armeniso* (BI, 2 sheets); gariga a *Sideritis sicula* al Km 3-4 sulla via del Carpino (Gargano); 5/VI/1952, *Corti* (BI); Murge di Altamura (Bari), 20/V/1943, *s.coll.* (BI); Bosco Luciani, Acquaviva delle Fonti, 25/V/1953, *E. Salazzo* (BI); Lecceta tra Manduria e S. Pietro, 23/V/1950, *Corti* (BI); Pulo di Altamura, 23/V/1953, *s.coll.* (BI);

Gargano, S. Giovanni Rotondo, querceto (ceduo) sopra a Val Carbonara (fianco sud) verso Masseria Corvara, 4/VI/1952, *Messeri* (BI); Bosco Marinelle, 27/V/1949, *C. Sallustio* (BI); Murge di S. Elio, 18/VI/1949, *F. Scaramozzi* (BI); Quasano, Bosco Sentinella, 12/VI/1950, *F. Scaramozzi* (BI); Gioa del Colle, Masseria Bosco, 3/V/1953, *M. Lingalone* (BI); Barletta, V/1847, *Bruni* (PORUN); Valle della Torre a sud di Borgo Celano (San Marco in Lamis), gariga con *Stipa austroitalica* con abbondante rocciosità e petrosità (calcari), 510 m, 15/V/2009, *R.P. Wagensommer* (Herb. Wagensommer); nei pressi di San Marco in Lamis (San Marco in Lamis, Foggia), 551525, 4618919, ED50 33T, pascoli aridi su terra rossa, 507 m, 9/VII/2007, *F. Bartolucci* (APP, 9 sheets); Contrada Olmo-Ciglie (Brindisi), incolto, 27/V/1991, *C. Bellanova* (LEC); Brindisi, incolto, 20/III/1990, s.coll. (LEC); S. Susanna (Brindisi), prato arido, 4/V/1997, *A. Albano* (LEC); Trochiarolo (Brindisi), bordo strada, 3/VI/1991, *G. Tafuro* (LEC); Mass. Mattarello-S. Pancrazio (Brindisi), incolto, 26/V/1991, *G. Avantaggio* (LEC); tra Siponto e Manfredonia (Siponto, Foggia), 24/V/1968, *F. Tammaro* (AQUI); sotto M.te S.Angelo (Foggia), gariga, 27/V/1968, *F. Tammaro* (AQUI); M. S. Angelo (Foggia), pascoli aridi, 26/V/2007, *s.coll.* (AQUI); Lizzanello (Lecce), incolto, 3/VI/1991, *s.coll.* (LEC); Villa Tresca (Monteroni, Lecce), incolto, 3/V/1991, *s.coll.* (LEC); *ibidem*, 20/V/1991, *F. Marra* (LEC); *ibidem*, 14/V/1990, *S. Manca* (LEC); *ibidem*, 22/IV/1989, *s.coll.* (LEC); *ibidem*, 18/V/1992, *s.coll.* (LEC); *ibidem*, 10/V/1988, *L. Biscozzo* (LEC); *ibidem*, 29/V/1990, *s.coll.* (LEC); *ibidem*, 30/V/1990, *s.coll.* (LEC); *ibidem*, 14/V/1990, *s.coll.* (LEC); *ibidem*, 29/V/1990, *s.coll.* (LEC); *ibidem*,15/V/1990, *R. Temeraro* (LEC); *ibidem*, 5/VI/1990, *G. Buoanno* (LEC); *ibidem*, 2/V/1990, *s.coll.* (LEC); *ibidem*, 8/V/1990, *s.coll.* (LEC); *ibidem*, 29/V/1990, *s.coll.* (LEC); *ibidem*, 28/V/1990, *s.coll.* (LEC); *ibidem*, 6/IV/1989, *Conazzo* (LEC); *ibidem*, *s.coll.* (LEC); *ibidem*, 19/V/1991, *F. Flores* (LEC); *ibidem*, 7/V/1990, *s.coll.* (LEC); *ibidem*, 27/V/1988, *Mele, Frugio* (LEC); *ibidem*, 14/IV/1989, *s.coll.* (LEC); Monteroni (Lecce), incolto, 26/V/1992, *s.coll.* (LEC); *ibidem*, 2/VI/1991, *S. Brandini* (LEC); *ibidem*, 2/VI/1991, *D. Leo* (LEC); *ibidem*; gariga, 18/V/1991, *s.coll.* (LEC); loc. Tre Pietre (Galatone, Lecce), gariga, 15/V/1996, *S. Leondura* (LEC); Zona "Tempi Nuovi" (Cavallino, Lecce), gariga, 27/IX/1985, *S. Scanduta* (LEC); Salice (Lecce), macchia, 6/V/1991, *s.coll.* (LEC); loc. Armino (Otranto, Lecce), incolto, 3/VI/1991, *D. Baccadamo* (LEC); Torre dell'Orso

(Lecce), incolto, 28/IV/1992, *s.coll.* (LEC); *ibidem*, 12/VI/1991, *D. Romito* (LEC); Lizzanello (Lecce), incolto, 24/V/1991, *s.coll.* (LEC); "Mater Domini" (Lecce), oliveto, 12/VI/1991, *A. Morleo* (LEC); Fiorini-Arnesano (Lecce), incolto, 5/V/1990, *s.coll.* (LEC); Macchia di Ponente (Montesano-Tricase, Lecce), 1/V/1991, *S. Marchiori* (LEC); Mass. Coni-Borgagne (Lecce), incolto, 22/V/1996, *P. Marasco* (LEC); Via S. Barbara (Copertino, Lecce), cava, 30/V/1996, *L. Martina* (LEC); strada per Arnesano (Lecce), incolto, 3/VI/1991, *Marconi* (LEC); S. Martino (Maglie, Lecce), muro a secco, 24/V/1991, *M. L. Nicita* (LEC); *ibidem*, 15/V/1991, *M. L. Nicita* (LEC, 2 sheets); Lecce, incolto, 18/V/1991, *C. Rosafio* (LEC); *ibidem*, 2/VI/1991, s.coll. (LEC); *ibidem*, 2/VI/1991, *G. Guerrieri* (LEC); *ibidem*, VI/1991, *A. M. Maria* (LEC); *ibidem*, 27/V/1990, *G. Guerrieri* (LEC); *ibidem*, 12/VI/1991, *s.coll.* (LEC); S. Pancrazio alla cava abbandonata (Lecce), gariga, 16/V/1996, *S. Scanduta* (LEC); Mondo Nuovo (Nardò, Lecce), incolto, 1/V/1989, *s.coll.* (LEC); Bella Greca (Scorrano, Lecce), margine strada, 5/V/1988, *Puce, Marchiori* (LEC); Lecce, […] erboso, 20/IV/1988, *Conte, Frugio* (LEC); Grottaglie nella Valle del Riscio (Lecce), s.d., *Lacaita* (FI); Otranto presso lo stagno Alimini (Lecce), calcare, 50 m, 31/V/1913, *A. Fiori* (FI); Statte (Taranto), incolto, 5/V/1991, *s.coll.* (LEC); Lizzano (Taranto), incolto, 13/IV/1991, s.coll. (LEC); Campomarino (Taranto), incolto, 5/VI/1991, *A. Pichierri* (LEC); ex aeroporto militare (Manduria, Taranto), prato arido, 9/V/1997, *A. Albano* (LEC); *ibidem*, 14/IV/1995, *A. Albano* (LEC); *ibidem*, 9/V/1997, *A. Albano* (LEC); Vallone del Pensiero (Grottaglie, Taranto), prato arido, 3/V/1997, *A. Albano* (LEC); Mass. Grazioli-Moruggio (Taranto), incolto, 27/III/1990, *G. Lomontice* (LEC); Masseria Leucaspide (Taranto), incolto, gravina, 3/VI/1992, *M. R. Spadaventia* (LEC); Campi Salati, incolto, 3/VI/1991, s.coll. (LEC); Capilungo (Marina di Alliste), incolto, 12/V/1989, *Conazzo* (LEC); Tarentum (Taranto), in saxosis aridis loco dicto Leucaspide, sol. calc., 50 m, VII/1907, *Sportelli V.* (FI, 2 sheets; NAP, P03886153, RO); in aridis apricis pascuis Monti Gargani circa M. S. Angelo, M. Sacro at p. sub 1500-2300, sol. calc., s. alt., 1/VI/1874, *Rigo, Porta* (NAP); M. Garganus […] in pascuis aridis circa M. Sant'Agelo, M. Sacro at p. 1-2200′ provenit etiam circa Alessano in Japygia, sol. calc., s. alt., 24/VI/1875, *G. Rigo* (NAP); Puglie presso Spinazzola, 8/V/1898, *A. Fiori* (RO); Grotta di S. Angelo (Taranto), VII/1963, *Parenzian* (RO); Murge tra Cassano,

Valantano, Locorotondo, Massafra, Laterza (S.B.I.), 27-30/IV/1975, *Anzalone* (RO); San Marco in Lamis, VI-VII/1927, *Giuliani* (RO); Andria \ Pulsano presso Taranto, 10/VI/1824, Casale \ *Gussone* (NAP); Trani, *s.d.*, *Gussone* (NAP); Manfredonia, 17/V/1840, *Gussone* (NAP); in pratis ad meridiem Barletta, IV/1844, *Bruni* (NAP, FI); Colline di Lecce, VI/1824, *Gussone* (NAP); Ugento presso il Capo di Leuca, 2/VI/1824, *Gussone* (NAP); Cerignola a S. Cassano, 15/V/1840, *Gussone* (NAP); in collibus apuliae, *s.d.*, *Tenore* (FI); Valle di Riscio presso Grottaglie (Terra d'Otranto), 11/VI/1919, *Lacaita* (FI); Taranto a Leucaspide, VII/1907, *Lacaita* (FI); Macchie di San Giovanni (Taranto) in calcareis aridissimis, c. 50 m, 1/VII/1914, *Lacaita* (FI); Italia Austral. Apulia. Gargano in pascuis erectis M.tis S. Angelo e M. Sacro, sol. calc., 1500-2000, 28/VI/1875, *Porta, Rigo* (FI, 5 sheets); Leucaspide, VI/1883, *Vitantonio* (FI); presso il Canale san Nicola, Macedonia, Japigia, VI/1807, *Profeta* (FI); dintorni di S. Nicandro, 17/V/1893, *U. Martelli* (FI); in collibus aridis Leucaspide prope Tarentum, IV/1811, *Profeta* (FI); Leucaspide prope Taranto, in sax. calc. aridi, c. 40 m, VII/1907, *C. Lacaita* (P04061752); Foggia, 11/VI/1917, *G. Paoli* (FI); Bosco […] alla Murgia di Tassitto/incolto del Vallone tra Rutigliano e Pacifico, 14/VI/1896 / 14/V/1896, *Palanza* (FI); Valle Fratta presso M. S. Angelo, VI/1896, *Martelli* (FI); Italia Meridional., Apulia in pascuis erectis M. S. Angelo, sol. calc., 1874, *Porta, Rigo* (FI); Puglie preso Spinazzola, 8/VI/1898, *A. Fiori* (FI); dintorni di S. Nicandro, 13/V/1893, *Martelli* (FI); Gargano: M. Croce, 12/VII/1915, *A. Fiori* (FI); Tarentum (Taranto), in saxosis aridis loco dicto Leucaspide, sol. calc., 50 m, VII/1907, *Sportelli V.* (FI, P03886153); Gargano al Monte S. Angelo, 14/VI/1898, *A. Fiori* (FI); Le Murgie, gariga presso la Masseria Rinaldin lungo SS 170 a SO di Minervino Murgie, 500 m ca, 11/VI/1968, *E. Nardi, R. Bavazzano* (FI); rocce calcaree della gravina di Gravina di Puglia, 300-330 m, 12/VI/1968, *E. Nardi, R. Bavazzano* (FI); gariga a *Sideritis sicula* presso S. Marco in Lamis, 650 m, 29/V/1968, *G. Moggi* (FI); gariga e rocce della Gravina di Laterza (Taranto), 100-300 m ca, 13/VI/1968, *E. Nardi, R. Bavazzano* (FI); Le Murgie, gariga e rocce della Murgia di Lamapera a NO di Gravina di Puglia, 688 m ca, 12/VI/1968, *E. Nardi, R. Bavazzano* (FI); rocce calcaree lungo il Bivio "La Cavola" e M.te s. Angelo, esp. N, 10/VI/1968, *E. Nardi, R. Bavazzano* (FI); Gariga a *Sideritis sicula* in Valle Carbonara (M. S. Angelo), 600-650 m,

26/V/1968, *G. Moggi* (FI); Grottaglie, XI/1916, *M. Guadagno* (2 sheets) (PI); Gravinola (Taranto) in saxosis calcareis, 18/III/1906, C. *Lacaita* (PI); Leucaspide (Taranto) in saxosis aridissimi ubi copiosissima, VII/1907, C. *Lacaita* (PI); Leucaspide prope Taranto, in sax. calc. aridi, ca 40 m, *C. Lacaita* (P04061752); Apulia-Tarentum (Taranto) in saxosis aridis loco dicto Leucaspide solo calcareo, alt. 50 m, VII/1906, *V. Sportelli* (PI); Gravina di Campalato (Gargano), 5/VI/1982, *Brullo, Signorello* (CAT); rupi sopra Manfredonia (Gargano), 5/VI/1982, *Brullo, Signorello* (CAT); tra M. S. Angelo e Manfredonia, 6/VI/1982, *Brullo, Signorello* (CAT); versante sud di Monte Tre Titoli (Accadia, Foggia), cerreta, pascoli, rupi, 750-930 m, 27/V/2011, *F. Conti, F. Bartolucc*i (APP Nos 46477, 46695 [epitype])*; Le Serre (Deliceto, Foggia), margine stradale, boscaglia di Robinia pseudacacia e roverella s.l., ambienti umidi, stipeto a *Stipa austroitalica*, 620-850 m, 26/V/2011, *F. Conti, F. Bartolucci* (APP No. 46490)*; Gargano, prima di San Marco in Lamis, presso il paese (San Marco in Lamis, Foggia), garighe e pendii aridi, 600 m, 03/VI/2016, *F. Bartolucci, V. Impiccini* (APP No. 57760); Lamalunga (Santeramo in Colle, Bari), prateria a *Stipa austroitalica*, 465 m, 12/05/2018, *F. Conti & F. Bartolucci* (APP Nos 60989-60991); Gravina del Triglio (Statte, Taranto), praterie, cespuglieti, vegetazione sinantropica, 148 m, 10/05/2018, *F. Bartolucci & F. Conti* (APP No. 61111); Masseria Donnarosa (Laterza, Taranto), bosco a *Quercus trojana* con *Raponticoides centaurium*, *Dictamnus albus*, *Paeonia mascula*, 395 m, 11/V/2018, *F. Conti & F. Bartolucci* (APP No. 61126); Gravina di Laterza (Laterza , Taranto), garighe a *Thymbra capitata*, rupi con *Piptatherum holciforme*, vegetazione casmofitica con *Campanula versicolor*, *Aurinia saxatilis megalocarpa*, *Scrophularia lucida*, 328 m, 11/V/2018, *F. Conti & F. Bartolucci* (APP No. 61203); presso agriturismo Murà (Altamura, Bari), praterie aride, 494 m, 04/VI/2016, *F. Bartolucci, V. Impiccini* (APP); nei pressi di Monte Sant'Angelo (Foggia), praterie aride, 494 m, 03/VI/2016, *F. Bartolucci, V. Impiccini* (APP); Santeramo in Colle (Bari), presso Iazzo Torretta (WGS84 40.7555°N, 16.73049°E), stipeto a *Stipa austroitalica*, 453 m, 14/V/2025, *F. Bartolucci* (APP); Altamura (Bari), loc. Scalcione (WGS84 40.8548°N, 16.61715°E), stipeto a *Stipa austroitalica*, 405 m, 13/V/2025, *F. Bartolucci* (APP); Altamura (Bari), presso Masseria Chinunno (WGS84 40.88067°N, 16.66914°E), prateria a *Stipa austroitalica*, 462 m, 14/V/20225, *F. Bartolucci* (APP); Andria (bari),

presso Piano di Citulo (WGS84 40.0589°N, 16.22934°E), stipeto a *Stipa austroitalica*, 518 m, 12/V/2025, *F. Bartolucci* (APP); **Basilicata**. Potenza (Lucania), estate 1912, *Gargiulo* (NAP); Matera (Lucania), 8/V/1911, *Armenante* (NAP); Nova Siri (Lucania), 28/VI/1907, *Cavara* (NAP); Pizzuto di S. Michele (Vulture), s.d., *Terracciano* (NAP); Lucania: Lavello alle Coste, 16/V/1845, *Gussone* (NAP); *ibidem*, 16/V/1846, *Gussone* (NAP); Melfi sulle colline, V/1860, *Terracciano* (NAP); Potenza in aridis saxosis loco dicto Costa di Centomane, solo calcareo, 750 m, 6/VI/1923, *Gavioli* (FI); Potenza in pascuis loco dicto la Botte, solo calcareo, c. 900 m, VI/1910, *Gavioli* (FI); *ibidem*, 7/VI/1923, *Gavioli* (FI); Potenza in pascuis aridis montis Ciciniello, c. 900 m, 2/VII/1923, *Gavioli* (FI); *ibidem*, 29/VI/1932, *Gavioli* (FI); *ibidem*, 700-900 m, 19/VI/1927, *Gavioli* (FI); Potenza, Monte Montocchio, paturages calcaires, 1160 m, 28/VI/1933, *Gavioli* (FI, P04061753, P04061754, P04436382, P04436383); Potenza in pascuis loco dicto Costa della Goveta, solo calcareo, c. 900 m, VI/1905, *Gavioli* (FI); Pietrapertosa (Basilicata) in lapidosis prope oppidum c. 1050 m, solo schistoso-arenaceo, 14/VI/1910, *C. Lacaita* (FI); strada Gravina-Irsina, cresta sinistra del Basentello (Spinalva) sopra la cantoniera, 16/V/1950, *Negri, Messeri* (FI); Pignola in herbidis loco Campo di Giorgio vocato, solo argilloso, 900 m, 19/VI/1927, *Gavioli* (FI); Picciano (Matera), querceto, 23/V/1951, *R. Corti, E. Franoini, G. Negr*i (FI); Difesa Melodia (Matera), 22/V/1951, *R. Corti, E. Franoini, G. Negri* (FI); Masseria Tarantini Difesa Melodia (Matera), 16/V/1950, *Negri, Messeri* (FI); Lucignano (Selva di Venusio) (Matera), bosco a *Quercus macedonica*, 22/V/1951, *R. Corti, E. Franoini, G. Negri* (FI); prope Potentiam, VI/1880, *Biondi* (FI); sul valico di Spinalva (Strada Gravina-Irsina) versante che guarda Gravina, 16/V/1950, *Negri, Messeri* (FI); in collibus aridis Melfi, VI/1860, *Terracciano* (FI); Abriola Serra di Monte Forte, in herbosis, alt. 1450, solo calcareo, 19/VI/1927, *Gavioli* (FI); **Basilicata**/**Apulia**. Basilicata-Lecce, Herb. *Tenore s.n.* (NAP, lectotype, bottom-right specimen); **Calabria.** Gizzeria prov. Catanzaro (Catanzaro), 630 m, 12/V/1996, *D. Puntillo* (CLU); Petrosa (Massiccio del Pollino, a Nord di Castrovillari, Calabria) (Castrovillari, Cosenza), prato, sustr. calcareo, esp. Sud Sud-Ovest, 650 m, 4/VI/1996, *L. Bernardo, N. Passalacqua, M. Aversa, A. Beni* (CLU, 3 sheets); Muraglione Cassano allo Ionio (Cosenza), 590 m, 15/V/1993, *A. Capparelli, Bernardo L.* (CLU); Morano Calabro le long de la route nationale n° 19 au Km

190 (Cosenza), bord du paturage en terrains calcaires a exposition est, alt. env. 750 m, 10/VI/1997, *G. Aldobrandi, R. M. Baldini* (FI); Eianina (Cosenza), 500 m, 23/V/1985, *F. Conte* (CLU); Colle Moschereto (Massiccio del Pollino, a Nord di Castrovillari Calabria) (Castrovillari, Cosenza), pascolo sassoso, roccioso, esp. Nord, 1300 m, 9/VII/1996, *L. Bernardo, N. Passalacqua, M. Aversa, A. Beni* (CLU); Monte Pollino, VII/1880, *Biondi* (FI); Monte Pollino: in pascuis aridis montanis, solo calc., 600-1000 m, s.d., *Rigo* (FI); in pascuis montis "La Dirupata", calc., 800 m, 15/VII/1907, *G. Rigo* (NAP); M. Pollino, in pascuis aridis montanis, sol. calc., 600-1000m, 22/VI/1898, *G. Rigo* (NAP, PI); in aridis ad Pollino […], s.d., *Baldacci* (RO); Torre di Giorgio, VII/1889, *Terracciano* (RO); Discesa di Morano, Calabria, 7/VI/1827, *Gussone* (NAP); sul Pliocene Laino, 26/VI/1917, *M. Guadagno* (PI); Faggeti a Campolongo gruppo del Cozzo del Pellegrino, 1200 m, 26-27/VII/1917, *M. Guadagno* (PI); tra Saracena e Campolongo, 26-27/VI/1917, s.coll. (PI); Pollino tra Civita e Frascineto, 600-700 m, 24/VI/1918, *M. Guadagno* (PI, 2 sheets); Monte Pollino, VII/1880, *Biondi* (FI); Massiccio del Pollino, lungo la SS 19 nei pressi di Morano Calabro (Morano Calabro, Cosenza) (ED50 33S 595415 4411809), pascoli aridi su calcare, 732 m, 8/VI/2008, *F. Bartolucci* (APP, 10 sheets); **Sicily**. in aridis calcareis montosis elatis Modonie serre di Quacedda, VII/1877, *M. Lojacono Pojero* (G-BU); Monte Stagnataro (Santo Stefano di Quisquina, Agrigento), incolti, 1000 m, 7/VI/1985, *Romano S., Ottonello D.* (PAL); loc. Quacella, Madonie (Polizzi Generosa, Palermo), suolo calcareo, 1200 m, 25/VII/2003, *Certa G., Scafidi G., Schimmenti E.* (PAL, 2 sheets); Riserva dello Zingaro (Trapani), incolti, 15/V/1984, *Mazzola P., Romano S.* (PAL, 4 sheets); Pres du torrent Vaccarizzo aproximite du fleuve Salso, a cote de l'autoroute Palerme-Caltanissetta, Sicilia centrale, 410 m, 27/V/1995, *L. Gianguzzi, G. Certa* (PAL); Alcamo, *s.d.*, […] (PAL, 2 sheets); Madonie, *s.d.*, […] (PAL); Castrogiovanni, Caltanissetta, s.d., […] (PAL); Caltanissetta, VI/1890, […] (PAL); Militello, s.d., […] (PAL); Etna, s.d., […] (PAL, 3 sheets); Milo Faito, Monte delle Feste, VI/1829, […] (PAL); Piano della Battaglia di Petralia, VI/1888, *H. Ross.* (PAL); Madonie, s.d., s.coll. (PAL); Peloritami, M. Scuderi, 38° 03' N, 15° 24' E, calcareous soli, 800-900 m, 13/VI/1990, *Raimondo F.M.* et al. (PAL); Sicani: Monte Rose, 37° 39' N, 13° 25' E, carbonatic soil, 1000-1200 m, 1/VI/1990, *Raimondo*

*F.M.* et al. (PAL); in rupes calcareis Busambra, s.d., s.coll. (PAL); […] tra Militello e Grammichele [...], s.d., s.coll. (PAL); Polizzi vicino il Paese, 10/VI/1847, s.coll. (PAL); Bosco a San Vito, s.d., *Todaro* (PAL); in apricis montanis […] del Bosco […], s.d., Lojacono (PAL); Busambra, VII, *M. Lojacono* (PAL, 2 sheets); Sicilia, s.d., *Gussone* (RO); Palermo in pasuis montosis, s.d., *Reina* (RO); Scopello, s.d., *Gussone* (NAP, 3 sheets); Caltanisetta, V, *Gussone* (NAP); Colline di Maletto verso Bronte, s.d., *Gussone* (NAP); Castrogiovanni, V, *Gussone* (NAP); Caronia, s.d., *Gussone* (NAP); Sicilia a Scopello, s.d., *Gussone* (NAP); Nicosia, V, *Gussone* / Amorosa a Sud di Busambra, s.d., *Gussone* / Mannatrini, s.d., *Gussone* (NAP); Madonie, s.d., *Gussone* (NAP); Sicilia a Scopello, s.d., s.coll. (NAP); in aridis montosis 1 / Madonie 2, Luglio (2), *Gussone* (NAP); Madonie a […] vicino il Passo della Botte, s.d., *Palatore* (FI); in Sicilia a Castrogiovanni, s.d., *Palatore* (FI); Sicilia, s.d., *Sorrentino* (FI); in montosis Polizzi, VII/1880, *M. Lojacono Pojero* (FI); in arido montano Marianopoli (Sicilia), VI, *M. Lojacono Pojero* (FI); Nicosia, *s.d., s.coll.* (FI); Madonie sopra Polizzi, s.d., *Parlatore* (FI); Sicilia, *s.d., s.coll.* (FI); Marianopoli, ad calcarea subelata, *Tornabene* (CAT); in rupestribus Randazzo, 1000 m, VI/1909, *Zodda* (CAT); M. S. Salvatore, 21/VII/1979, *S. Brullo* (CAT, 2 sheets); Madonie, 3/IX/1972, *S. Brullo* (CAT); *ibidem*, 3/VIII/1982, *S. Brullo* (CAT); base del Monte Quacella (Madonie), 31/V/1986, *Brullo, Minissale* (CAT): Sorgente Furma la Cara (Piazza Armerina), s.d., *Galesi* (CAT); Tre Grotte, Piana di Buccheri, s.d., *Galesi* (CAT); Castrovillari, s.d., *Galesi* (CAT); strada tra Centuripe e Catenanuova, 18/V/1987, *Minissale* (CAT);); in aridis calcar. Bisacquino, VI/1903, *M. Lojacono Pojero* (G-BU); in aridis calcar. Bisacquino, VI/1903, *M. Lojacono Pojero* (G-BU).

***Thymus striatus*** Vahl subsp. ***striatus***
**Lazio:** Punta dell'Uccettu, (Borgorose, Rieti), rupi, 1972 m, 8/VI/2009, *F. Bartolucci* (APP, 4 sheets)*; Montagna della Duchessa in loc. Coppo dei Ladri, (Borgorose, Rieti), pascoli, 1580 m, 28/VI/2008, *F. Bartolucci* (APP, 6 sheets); Montagna della Duchessa: M. Morrone vers. SW, (Borgorose, Rieti), pascoli sassosi, su calcare, 2000 m, 15/VII/2008, *F. Bartolucci* (APP, 5 sheets)*; Monti Simbruini M. Viglio (1650-1850 m), 23/VII/1995, *Anzalone* (RO); **Abruzzo:** M. Cefalone (Lucoli, L’Aquila), 19/VII/2023, *F. Conti, F. Bartolucci, G. Cangelmi*

(APP Nos 67431, 67432); parete verticale alla base della Cima delle Murelle, (Pennapiedimonte, Chieti), rupi, 2188 m, 30/VII/2009, *F. Bartolucci* (APP, 5 sheets)*; *ibid.*, 2300 m, 7/VIII/2011, *F. Bartolucci* (APP, 4 sheets); Monte Rozza, loc. Saravastrello, (L'Aquila), pascoli aridi, 1328 m, 29/VII/2008, *F. Bartolucci* (APP); Monte Rozza, loc. Iacci di Rozza, (L'Aquila), pascoli aridi, 1891 m, 29/VII/2008, *F. Bartolucci* (APP, 3 sheets); Gobbe di Selva Romana (Pennapiedimonte), pascoli aridi, 1800 m, 28/07/1991, *F. Conti* (APP No. 13000)*; Campo Imperatore - Valle Cortina (Calascio), 42°24' 11''E, 13°42' 21''N, UTM-ED50, pascoli sassosi calcarei, 1550 m, 29/VI/2002, *F. Conti & al.* (APP No. 5568)*; nei pressi del Rifugio S. Elia (Collelongo, L'Aquila), pascoli aridi, 1619 m, 15/VI/2010, *F. Bartolucci, N. Ranalli* (APP, 7 sheets)*; Serra Lunga (Villavallelonga, L'Aquila), pascoli e faggeta, 1759 m, 15/VI/2010, *F. Bartolucci, N. Ranalli* (APP No. 50148); dallo Scoppaturo a Piana dell'Ospedale (Castel del Monte, l'Aquila), rupi, 19/VII/2013, *A. Stinca, F. Bartolucci F. Conti* (APP Nos 53021, 53024*); Valle Sevice, (L'Aquila), pascoli aridi, 18/VII/2005, *F. Bartolucci* (APP); **Molise**: Pescopennataro (Pescopennataro, IS), 1142 m, 10/06/2006, *F. Conti* (APP No. 64727); **Campania:** Mandranello (Padula, Salerno), pascoli, 1060 m, 06/VI/2013, *F. Conti & F. Bartolucci* (APP Nos 52467, 52469*); Mandrano (Padula, Salerno), pascoli aridi, 1030 m, 06/VI/2013, *F. Conti & F. Bartolucci* (APP No. 52496)*; Avvocata di Majori, c. 3500′, in loose soil, forma laxior in solo pumiceo, 29/VI/1883, *C. Lacaita* s.n. (BM, 3 sheets); Monte S. Angelo di Castellammare, in cacumine, 1448 m, 10/07/1907, *C. Lacaita* s.n. (BM); Valle Agricola (Matese) (Caserta), V/2007, *L. Peruzzi* (APP, 2 sheets)*; sentiero per Monte Cappello, (Caserta), prato arido, s.d., *s.coll.* (IS); Monte Bulgheria, (S. Giovanni a Piro, Salerno), 970-1200 m, 15/VI/1993, *L. Bernardo* (CLU); prov. di Salerno: in rupestribus montium dictorum dell'Avvocata di Majori, (Salerno), solo calcareo, 900 m circ., 28/V/1910, *C. Lacaita* (FI, 3 sheets; NAP; RO); M. S. Angelo di Castellammare, (Salerno), s. calc., 1400 m, VII/1911, *M. Guadagno* (FI, 2 sheets); M. S. Angelo di Castellammare, (Salerno), s. calc., VII/1911, *M. Guadagno* (FI); qua e la sul Monte S. Michele, (Salerno), sui 1000 m, 30/VIII/1949, *s.coll.* (FI); S. Maria a Castello, (Vico Equense, Salerno), s. calc., 700 m, 25/V/1913, *M. Guadagno* (FI); S. Angelo a Castellammare, (Salerno), c. 3500, s.d., (da Pasquale in Luglio 1830) (FI); Avvocata di Majori, c.

3500 between Salerno e Amalfi, (Salerno), 28/VI/1883, *C. Lacaita* (FI); M. S. Angelo di Castellammare in cacumine, (Salerno), c. 4900, 26/VI/1883, *C. Lacaita* (FI); M. S. Angelo a Tre Pizzi (Costiera Amalfitana) (Castellammare di Stabia, Salerno), pascoli, 1453 m, 21/VI/2008, *L. Cancellieri* e *G. Salerno* (APP, 7 sheets); M. Faito di Castellammare, nei pressi del Convento di S. Michele, (Castellammare di Stabia, Salerno), pascoli, 1200 m, 3/VI/2008, *F. Bartolucci* (APP, 15 sheets)*; Col di Lammardi, gruppo del M. Cervati (Sanza, Salerno), 12/VII/2004, *E. Del Vico* (APP); M. Motola, (Teggiano, Salerno), 10/VI/2006, *E. Del Vico* (APP); M. Cervati, (Salerno), 7/VII/2007, *G. Ciaschetti* (APP); Monte Polveracchio, (Salerno), saxosis cacumine, 1790, VII/1902, *Lacaita* (PAL); In Monte S. Angelo prope Castellamare, solo calcareo, 1/VII/1873, *P.G. Strobl* (WU); Cilento, 4/VII/2004, *E. Del Vico* (APP); *ibidem*, 5/VII/2004, *E. Del Vico* (APP); *ibidem*, 8/VII/2004, *E. Del Vico* (APP, 3 sheets); *ibidem*, 2/VII/2004, *E. Del Vico* (APP); Monte Vergine (in hirpiniis), 15/V/1909, *Grande* (NAP); Monti di Castellammare (prope Neapolim), 14/V/1908, *Grande* (NAP); in montibus stabianus loco dicto "Faito", in rupestribus calcareis aridis, 1100 m, 14/VI/1908, *Grande* (NAP); Monte Bulgheria (Cilento), 9/V/1912, *Grande* (NAP); Matese all'Esule, 8/VII/1914, *Cavara* & *Grande* (NAP); S. Maria a Castello, (Vico Equense), sol. calc., 700 m, 15/V/1913, *Guadagno* (NAP); Monti di Castellammare, VI/1906, *Cavara* (NAP); Monte Vergine (in hirpiniis), 15/V/1909, *Grande* (NAP); Monte Falesio di Majori (Majori), in saxosis cacumine, c. 1100 m, VII/1881, *Lacaita* (PAL); M. S. Angelo a Tre Pizzi, 1440 m, VII/1902, *Lacaita* (PAL); Vetta del M. Mai, 1650 m, 19/VII/1921, *C. Lacaita* (PI); M. Mai, circa 1500 m, 19/VII/1921, *C. Lacaita* (PI); M. S. Angelo di Castellammare rupi sopra l'acqua Santa, VII/1911, *M. Guadagno* (PI); M. S. Angelo alla vetta e precisamente coll'Helianthemum candidissimum salendo dall'acqua Santa, VII/1909, *M. Guadagno* (PI); rupi sopra l'acqua Santa, 13/VII/1911, *M. Guadagno* (PI); rampe di Faito […], 700 m, V/1911, *M. Guadagno* (PI); M. S. Angelo di Castellammare al valico dell'acqua Santa, 20 /VII/1913, *M. Guadagno* (PI); M. S. Angelo di Castellam. rupi della vetta e non sopra l'acqua Santa, 13/VII/1911, *M. Guadagno* (PI); prov. di Napoli in Monte Faito di Castellammare, solo pumiceo prope locum dictum Porta di Faito, 1220 m, 26/VI/1924, *C. Lacaita* ex Flora Italica Exsiccata, series III n. 2952

(neotype G, isoneotypes CAT, FI, 3 sheets, PI, RO); Monti di Castellammare, 5/VII/1834, *Gussone* (NAP); Monti di Castellammare, 5/VII/1834, *s.coll.* (NAP); Monti di Castellammare, 20/VI/1835, *s.coll.* (NAP); S. Michele a Castellammare, 23/VI/1833, *s.coll.* (NAP); Matese alla Gallinola, 21/VII/1833, *Chiovitti* (NAP); Monti Stabiani (presso Napoli), s.d., *Grande* (FI); Monte Cervialto, 1400-1600 m, 9/VII/1974, *B. Moraldo* (FI); Monte Terminio: rupi Cannella, esp. S SW, 1300-1500 m, 9/VIII/1974, *B. Moraldo* (FI); Monte S. Angelo di Castellammare, rupi calcaree, 1400 m, 18/VII/1910, […] (FI); Monte S. Angelo di Castellammare, rupi calcaree, 1400 m, 18/VII/1910, […] (FI); Monte S. Angelo, (Castellammare), 1400 m, VII/1903, *M. Guadagno* (FI); M. S. Angelo di Castellam. rupi sopra l'acqua Santa, 13/VII/1911, *M. Guadagno* (PI); M. S. Angelo di Castellammare verso la cima del molare, VII/1903, *M. Guadagno* (PI); M. S. Angelo di Castellammare, s.d., s.coll. (PI); S. Maria a Castello, 700 m., 25/V/1913, *M. Guadagno* (2 sheets) (PI); S. Maria a Castello, 25/V/1913, *M. Guadagno* (PI); S. Maria a Castello con gli altri, s.d., s.coll. (PI); vetta di Monte Mai tra Calvanico e Fittone, (Calvanico, Salerno), c. 1600 m, 19/VII/1921, *C. Lacaita* (FI); **Basilicata:** Pozzi (Brienza, Potenza), prato arido, 1010 m, 05/VI/2013, *F. Conti & F. Bartolucci* (APP Nos 52315, 52320)*; Serra di Crispo, versante Lucano del Massiccio del Pollino, (Potenza), rupi, 2000-2050 m, 13/VIII/1992, *L. Bernardo, N. Passalacqua* (CLU); Sirino (Potenza), *s.d., G. Ciaschetti* (APP); Brutium M. Dolcedorme (Pollino), in rupibus, c. 2200, 5/VIII/1929, *O. Gavioli* (FI); M. Serra delle Ciavole (Pollino), in rupestribus calcareis, 2050 m, 4/VIII/1929, *O. Gavioli* (FI); in Lucania, s.d., Crocchi (PAL); Pizzo dei Corni Monte Sacro di Novi Lucania, 1600 m, 13/VII/1907, *M. Guadagno* (PI); Monti di Balvano, s.d., *Barbazita* (NAP); Basilicata, s.d., *Barbazita* (NAP); Monti della Lucania, 1839, *Barbazita* (NAP); Sirino, M. del Papa, vers. E (Potenza), pendii rupestri calc., 1800-2000 m, 25/07/1999, *F. Conti, D. Lakusic & Ph. Küpfer* (APP No. 1344); Sirino, M. del Papa, vers. W (Potenza) praterie sassose, 1900-2000 m, 25/07/1999, *F. Conti, D. Lakusic & Ph. Küpfer* (APP No. 1406); Serrone (Savoia di Lucania, Potenza), gariga a Salvia officinalis, 600 m, 07/06/2013, *F. Conti & F. Bartolucci* (APP No. 52340); Monte Pierno (San Fele, Potenza), rupi calcaree, 1075-1268 m, 05/06/2015, *F. Conti, F. Bartolucci, R. Pennesi* (APP Nos 55587, 55588); Monte Pierno (San Fele, Potenza), pascolo, 1075-1268 m, 05/06/2015, *F. Conti, F.*

*Bartolucci, R. Pennesi* (APP No. 55602); **Basilicata/Calabria.** Belvedere di Malvento sul versante sud di Timpone Capanna, Massiccio del Pollino, (Potenza/Cosenza), 1600-1650 m, 3/VII/1991, *L. Bernardo* (CLU); Serra del Prete, lungo la cresta verso la cima Massicio del Pollino, (Potenza/Cosenza), 2000-2160 m, 3/VII/1991, *L. Bernardo* (CLU); Belvedere di Malvento sul versante sud di Timpone Capanna, Massiccio del Pollino, (Potenza/Cosenza), 1600-1650 m, 3/VII/1991, *L. Bernardo* (CLU); **Calabria.** vetta del Dolcedorme (Pollino), 2300 m, 24/VIII/1918, *Guadagno s.n.* (PI No. 353_5768); Massiccio del Pollino, ca. 8,5 Km NW of Morano, Colle Anticristo SW of Cozzi Anticristo, (Morano Calabro, Cosenza), 1330 m, 20/VI/1997, *Partecipanti VIII Iter Mediterraneum* (CLU); Dolcedorme, 2100 m circa, 26/VIII(1908, *Cavara* e *Grande* (NAP); M.te Cocuzzo (Catena Costiera), cima, (Cosenza), sfatticcio di cresta, 1540 m, 2/VII/1989, *L. Bernardo, G. Cesca, P. Gallo, M. Codogno* (CLU); Dirupata di Morano Calabro, (Morano Calabro, Cosenza), 722m, 8/V/1994, *P. Calvosa* (CLU); Piano Pallone (vers. merid. Massiccio del Pollino), (Castrovillari, Cosenza), pascolo sassoso, substr. Calcareo, esp. O, 1535 m, 19/VII/1997, *L. Bernardo, N. Passalacqua,* M. Aversa, A. Beni (CLU); Piano Pallone ( vers. merid. Massiccio del Pollino), (Castrovillari, Cosenza), pascolo sassoso, substr. Calcareo, giac. N-S, 1525 m, 19/VII/1997, *L. Bernardo, N. Passalacqua, M. Aversa, A. Beni* (CLU); Colle Moschereto (Massiccio del Pollino, a Nord di Castrovillari Calabria), (Castrovillari, Cosenza), pascolo sassoso, roccioso, substr. Calcareo, esp. Nord, 1300 m, 9/VII/1996, *L. Bernardo, N. Passalacqua, M. Aversa, A. Beni* (CLU); Massiccio del Pollino, ca. 7 Km NNW of Morano, Colle Dragone, (Morano Calabro, Cosenza), 1100 m, 20/VI/1997, *Partecipanti VIII Iter Mediterraneum* (CLU); Timpa S. Lorenzo, (S. Lorenzo Bellizzi, Cosenza), 17/X/1993, *L. Bernardo* (CLU); Monte Mula, versante Nord sotto la cima, (S. Donato di Ninea, Cosenza), prato roccioso, 1870-1920 m, 22/VII/1991, *L. Bernardo, G. Cesca* (CLU); Monte Mula, cima, (S. Donato di Ninea, Cosenza), 1900-1935 m, 22/VII/1991, *L. Bernardo, G. Cesca* (CLU); Monte Mula, versante Nord sotto la cima, (S. Donato di Ninea, Cosenza), parti pingui e doline, 1900-1920 m, 14/VII/1994, *L. Bernardo, G. Cesca* (CLU, 3 sheets); Cozzo Pellegrino, (S. Donato di Ninea, Cosenza), prato cacuminale, 1970 m, 13/VII/1994, *L. Bernardo, N. Passalacqua* (CLU); Cozzo Pellegrino, (S. Donato di Ninea,

Cosenza), sfatticcio di cresta, 1980 m, 7/VII/1991, *D. Puntillo, L. Bernardo* (CLU); Valle del Fiume Argentino, lungo il sentiero per Fontana Massetti, (Orsomarso, Cosenza), 700-800 m, 28/V/1991, *L. Bernardo* (CLU); M.te Pollino (vers. meridionale del Massiccio del Pollino, Cosenza), rupi del vers. occ., Esp. W, 1900 m, 20/VII/1993, *L. Bernardo, N. Passalacqua* (CLU); Monte La Caccia, cima, (Belvedere Marittima, Cosenza), prato di cresta, 1700 m, 13/VII/1993, *L. Bernardo, N. Passalacqua* (CLU); Monte La Caccia, cima, (Belvedere Marittima, Cosenza), rocce di cima, esp. Sud, 1740 m, 13/VII/1993, *L. Bernardo, N. Passalacqua* (CLU); Monte Montea, (S. Sosti, Cosenza), seslerieto a *S. tenuifolia*, esp. W, 1750-1800 m, 19/VI/1994, *L. Bernardo, N. Passalacqua* (CLU); Serra del Prete,( Massicio del Pollino, a Nord di Castrovillari, Calabria), (Castrovillari, Cosenza), substr. Calcareo, 2000 m, 22/VII/1996, *L. Bernardo, N. Passalacqua* (CLU); Serra Dolcedorme, (Massiccio del Pollino), pendio pietroso, 2000-2160 m, 13/VIII/1992, *L. Bernardo, N. Passalacqua* (CLU); Monte Pollino (Cosenza): N of Morano Calabra just SE of Rifugio, limestone grassland, 1740 m, Akeroyd July Miles Rumsey, (FI); Monte Pollino, 1400-1500m, 11/VI/1877, Huter et Rigo (FI); Monte Pollino: pascoli intorno al Fortino a Nord di Campo Tenese, calcari e scisti, 1050-1100 m, 20/V/1972, Arrigoni Raffaelli et Bavazzano (FI); Serra del Prete, cresta di cima su sfatticcio, 2150 m, 19/VII/2006, *L. Peruzzi & G. Aquaro* (APP); creste della Montea, 26/VIII/1912, *Grande* (NAP); Cozzo Pellegrino Campolongo, 26/VI/1917, *M. Guadagno* (2 sheets) (PI); Cozzo Pellegrino vetta Cala a Campolongo, 1400 m, 26/VI/1917, *M. Guadagno* (PI, 4 sheets); Cozzo Pellegrino mulattiera tra Saraceno e Campolongo, 1000 m, 26/VI/1917, *M. Guadagno* (PI); Vetta del Dolcedorme (Pollino), 2300 m, 24/VIII/1918, *M. Guadagno* (3 sheets) (PI); Appennino Calabro-Lucano: Gruppo del M.te Pollino, Serra del Prete, garighe e pascoli del crinale si SO salendo dal Belvedere, garighe e pascoli, tra 1650 e 2000 m, 23/VII/1972, *E. Nardi, R. Bavazzano, Posca V.* (FI); Appennino Calabro-Lucano: Gruppo del M.te Pollino, gariga presso il Belvedere a SE del torrione della Capanna, gariga, 1600 m, 23/VII/1972, *E. Nardi, R. Bavazzano, Posca V.* (FI, 2 sheets); Appennino Calabro-Lucano: Gruppo del M.te Pollino, rocce calcaree e vallecole rupestri del crinale di SE tra q 2000 e la vetta del M. Pollino (m 2248) Esp. NE, rocce calcaree e vallecole rupestri, 2000-2248, 23/VII/1972, *E. Nardi, R. Bavazzano, Posca V.* (FI);

Monte Papa (Lagonegro), 2000 m circa, 19/VIII/1909, *Cavara* e *Grande* (NAP); in pascuis montis "La Dirupata", calc., 800-1200 m, 15/VII/1907, *G. Rigo* (NAP);

***Thymus striatus*** Vahl subsp. ***acicularis*** (Waldst. & Kit.) Ronniger
**ITALY. Emilia Romagna:** Monte Mauro, VI/1877, *Cortesi*? (FI); **Tuscany:** Monte Vaso (Chianni, Pisa), serpentino, 450-633 m, 23/05/2009, *F. Bartolucci & F. Conti* (APP No. 38542); Monte Vaso (Santa Luce, Pisa), gariga su serpentino, 580 m, 23/V/2009, *F. Bartolucci & F. Conti* (APP, 11 sheets)*; Monte Ferrato di Prato (Toscana), in fissuris rupium, 12/06/1908, *C. Lacaita* s.n. (BM, 2 sheets); Monte Ferrato di Prato (Toscana), in fissuris saxorum serpentinorum, 6/05/1906, *C. Lacaita* s.n. (BM); Monte Ferrato di Prato (Toscana), in fissuris rupium serpentinorum, 6/05/1906, *C. Lacaita* s.n. (BM); Toscana; Prov. di Firenze, Montignoso frequente sui gabbri, 17/VI/1911, *R. Pampanini* (BM); Etruria (Prov. di Firenze), Montaione, in rupestribus et silvaticis prope pagum Montignoso, in rupestribus et silvaticis, solo ophiolitico (gabbro), 17/VI/1911, *R. Pampanini* (FI, RO); M. Murlo, (Pieve Santo Stefano, Arezzo), rupi serpentinose, 415 m, 1/VI/2009, *F. Bartolucci* (APP, 10 sheets); Monte Ferrato, (Prato, Firenze), radura boschiva su serpentino, 204 m, 20/V/2009, *F. Bartolucci* (APP, 13 sheets)*; Monte Ferrato presso Prato, (Firenze), 11/VI/1910, *R. Pampanini* (FI); Montignoso, (Firenze), sui serpentini, 17/VI/1911, *R. Pampanini* (FI); serpentini dell'Impruneta, (Firenze), 4/V/1938, *Cei* (FI); *ibidem*, 23/VI/1939, *Cei* (FI); Impruneta, (Firenze), serpentini, 17/VI/1939, *I. Bargoni* (FI); *ibidem*, 24/V/1938, *I. Bargoni* (FI); Pomerance a M. Gabbri (Pisa), su serpentino, 350-380 m, 6/VI/1916, *A. Fiori* (FI); M. Gabbro sopra Bagni delle Galleraie (Pisa), 558 m ca, 29/VI/1953, *A. Chiarugi, O. Vergnano, R. Corradi* (FI); Podere di S. Apollinare, (Serrazzano, Pisa), serpentini, 550 m ca, 29/VI/1953, *A. Chiarugi, O. Vergnano, R. Corradi* (FI); M. Ferrato presso Prato, 14/VII/1910, *A. Fiori* (FI); Monte Ferrato (Figline) (Prato), 1/VII/1923, *A. Chiarugi* (FI, 2 sheets); M. Ferrato (Prato), 1/V/1934, *U. Losacco* (FI); M. Ferrato presso Prato, VI/1889, *s.coll.* (FI); Monte Ferrato presso Prato, 1/VI/1866, *s.coll.* (FI); Toscana (Firenze): Impruneta ai Sassi Neri, suolo serpentinoso, 315 m, 4/VI/1911, *A. Fiori* (FI)*; Etruria (Prov. di Firenze): copiosus in rupibus et saxis ophiolithicis montis Ferrato prope Prato, alt. 100-

200 m, 11/VI/1910 e 11/VI/1911, *R. Pampanini & A. Fiori* (FI, 3 sheets; RO, NAP); Alta Val Tiberina: Poggio delle Calbane, serpentino, 7/VI/1905, *Pichi Sermolli* (FI); Sassi Neri, Impruneta, 19/V/1935, *Losacco* (FI); Impruneta prope florentiam, in declivis aridis, solo serpentinoso (sui gabbri), 12/VI/1873, *S. Sommier* (FI); Radincondoli a M. Gabbri, serpentino, 550 m, 11/VI/1958, *A. Fiori* (FI); Pomerance entre Libbiano et Serrazzano, pres de Monterufoli, garrigue ser serpentine, a exposition s, 520 m, 17/V/2000, *P. Cuccuini & G. Aldobrandi* (FI); M. Ferrato, 8/II/1933, *G. Negri, A. Messeri, M. Corradi* (FI, 3 sheets); *ibidem*, 25/IV/1933, *A. Messeri* (FI); M. Ferrato, 2/V/1933, *A. Messeri, M. Corradi* (FI, 4 sheets); M. Ferrato presso Prato, 12/VI/1908, *Pichi Sermolli* (FI); M. Ferrato, serpentino, 12/VII/1933, *Pichi Sermolli* (FI, 2 sheets); Monte Ferrato, s.d., *Losacco* (FI); Alta Val Tiberina, Monte Petroso versante Ovest, serpentino, 25/VI/1935, *Pichi Sermolli* (FI); in rupibus ophiolithicis prope pagum Impruneta ad merid. Florentia, 12/VI/1973, *E. Levier* (FI); Impruneta sui gabbri, 5/V/1897, *S. Sommier* (FI); sulla cima di Monte Ferrato, 16/V/1873, *Gemmi* \ Monte Ferraro di Prato […],17/V/1874, ? (FI); **Umbria:** Monte […] Gubbio, 18/VII/1875, *Cherici* (FI); Costacciaro (Perugia), sentiero per lo Schioppo, 650 m, *E. De Santis* (APP)*; **Marche:** prov. di Forlì: Le Cete di Uffogliano, 20/V/1929, *R. Pampanini & R. Chiosi* (FI, 4 sheets); Monte Simoncello (Carpegna, Pesaro-Urbino), rupi, 1159 m, 14/VII/2018, *F. Conti, A. Stinca, R. Pennesi* (APP No. 58538); Fiume Candigliano (Fossombrone, Pesaro-Urbino), 364 m, 14/VII/2018, *F. Conti, A. Stinca, R. Pennesi* (APP No. 58550); Forca di Presta (Arquata del Tronto, Ascoli Piceno), 1530 m, 22/VII/2020, *F. Conti, F. Bartolucci* (APP No. 69578); Monte Ascensione (Rotella, Ascoli Piceno), rupe, 1103 m, 20/V/2009, *C. D'Angeli* (APP No. 39572); Pian Perduto, sotto il Rifugio C.A.I., Colle Minio (tutto il versante), esp. sud-sud-ovest, pascoli sus substr. calcareo, 1380-1450 m, 3/IX/2010, *S. Ballelli* (CAME); Forca di Presta, 16/VII/1856 \ 6/VII/1856, *Parlatore* (FI); Colle dei Mezzi Litri (M. Vettore, M. Sibillini, Ascoli-Piceno), roccia calcarea, 1670 m, 21/06/1994, *D. Puiatti* (ROV); Gola del Furlo, (Pesaro-Urbino), margine sentiero, ca. 330, 14/IV/2007, *L. Peruzzi* & *K. F. Carapelli* (APP); Monte Nerone, forra della Cornacchia, (Apecchio, Pesaro-Urbino), s.d., *G. Santoni* (APP); Monte di Amandola / Ascoli Pic., 30/VI/1886, *Silvestri* (RO); Monte Vettore, VII/1832, *Sanguinetti* (RO); Pioraco, stazioni rupestri,

VIII/1952, *Anzalone* (RO); Montefortino \ in radicibus Montis Sibillae […], VII/1840 \ VI/1841, *D. Marzialetti* (FI); […] Monte Catria tra 900 e 1000 m \ […] Catria […], 25/VII/1856, *Parlatore* \ *Piccinini* (FI); Monte di Amandola, 30/VI/1886, *Silvestri* (FI); Sasso di Simone app. del Monte Feltro, III/1876, *Cherici* (FI); **Lazio.** Vallunga, (Leonessa, Rieti), 860-920 m, 17/VII/1992, *L. Bernardo* (CLU); M. Navegna, (Rocca Sinibalda, Rieti), rupi, 1400 m, 10/VIII/2004, *F. Bartolucci* (APP); loc. Camparelli, (Castel di Tora, Rieti), pascoli aridi, 720 m, 19/V/2007, *F. Bartolucci* (APP); versante E del M. Navegna, (Varco Sabino, Rieti), pascoli aridi, 1300 m, 12/VII/2003, *F. Bartolucci* (APP); loc. F.te Raina, M. Navegna, (Rocca Sinibalda, Rieti), pascoli aridi, 1102 m, 6/VI/2002, *F. Bartolucci* (APP); M. Duchessa presso Cartore, (Borgorose, Rieti), pascoli aridi, 1000 m, 5/VI/2007, *F. Bartolucci* (APP)*; piano della Serra, (Roma), 11/VII/1974, *s.coll.* (AQUI); Tibur (Tivoli) in rupestribus et saxorum rimis, loco Acque Albule dicto, (Roma), sol. calcareo, 50 m, 17/V/1907, *L. Vaccari* (FI, 3 sheets; NAP); Tibur (Tivoli) in saxorum rimis prope lacum dei Tartari dictum, loco Acque Albule, (Roma), sol. calcareo, 50 m, VIII/1910, *A. Béguinot* (FI); Lago dei Tartari, travertini Acque Albule (Tivoli), 29-30/IV/1977, *Anzalone* (RO); Tivoli ai Bagni della acque albule, 22/V/1904, *L. Vaccari* (FI); Tivoli, Bagni (Roma), 20/V/1902, *L. Vaccari* (FI); Tivoli, Acque albule, 25/V/1884, *R. Pirotta* (FI); Montagna della Duchessa, 29/VIII/1952, *C. Steinberg* (FI); Solfatara di Tivoli, *s.d., s.coll.* (NAP); **Abruzzo:** Pizzoli (L'Aquila), pascoli aridi, III/2007, *F. Bartolucci* (APP); S. Venanzio (Raiano, L'Aquila), 30/VII/1970, *s.coll.* (AQUI); Macchialonga, prati di Cascina, esp N, (Cagnano Amiterno, L'Aquila), 1200, 27/VI/1975, *s.coll.* (AQUI); *ibidem*, ambienti a *Betula pendula*, 1200, 27/VI/1975, *s.coll.* (AQUI); Paganica (fossi), (L'Aquila), 620, 30/V/1968, *O. Ferella* (AQUI); Roio, (L'Aquila), 900, 11/V/1968, *C. Trionfi* (AQUI); Roio, (L'Aquila), *s.d., s.coll.* (AQUI); Gignano, (L'Aquila), 800 m, *s.d., s.coll.* (AQUI); lungo la strada tra Barisciano e S. Colombo, (Barisciano, L'Aquila), pendii rupestri, 1000 m, 16/VI/2004, *F. Bartolucci* (APP); al Conveto di S. Spirito d'Ocre, (Fossa, L'Aquila), pascoli aridi, 700 m, 17/V/2006, *F. Bartolucci* (APP, 6 sheets); loc. Costa Macere, tra Poggio Picenze e Barisciano, (Barisciano, L'Aquila), pascoli aridi, 1000 m, 17/V/2006, *F. Bartolucci* (APP, 6 sheets); Lago Sinizzo, (S. Demetrio ne' Vestini, L'Aquila), rupi, 710 m, 22/V/2007, *D. Di Santo* (APP); lungo la strada tra

Barisciano e S. Colombo, (Barisciano, L'Aquila), pendii rupestri, 1000 m, 19/V/2004, *F. Bartolucci* (APP); *ibidem*, 1000 m, 4/VI/2004, *F. Bartolucci* (APP, 3 sheets); S. Colombo, (Barisciano, L'Aquila), pascoli aridi, 1100 m, 10/VI/2004, *F. Bartolucci* (APP); *ibidem*, 28/VI/2005, *F. Bartolucci* (APP); nei pressi di Fossa Raganesca, (Ocre, L'Aquila), pascoli aridi, 830 m, 5/VI/2007, *F. Bartolucci* (APP, 5 sheets); pascolo argilloso e calcareo del versante sud di M. Pettino fortemente inclinato a sud, (L'Aquila), 750 m, 7/VI/1952, *Lusina* (RO); Montagna di Pettino e M. S. Giuliano, (L'Aquila), 9/VI/1987, *Anzalone* (RO); Colle di Ocre cava di Monticchio, (L'Aquila), 10/VI/1987, *Anzalone* (RO); Colle San Martino, (L'Aquila), 13/V/1920, *Parascenzo* (RO); Tremonti, (Popoli, Pescara), V/1968, *s.coll.* (AQUI); *ibidem*, luoghi aridi, 30/V/1969, *s.coll.* (AQUI); *ibidem*, 30/V/1968, *s.coll.* (AQUI); M. Piselli, (S. Giacomo, Teramo), pascoli aridi, 1300 m, 31/V/2006, *F. Bartolucci* (APP)*; Montagna dei Fiori, (Teramo), pascoli del v. SO, 1450-1480 m, 4/VII/1987, *G. Moggi, E. Luccioli, E. Tosi* (FI); Montagna dei Fiori tra S. Vito e Corano, (Teramo), pendio roccioso esp. SO sul versante interno, 900-1000 m, 27/V/1987, *G. Aldobrandi, G. Padovani, E. Tosi* (FI); Montagna dei Fiori, (Teramo), pascolo a SO del lago, 1620-1636 m, 15/VII/1987, *E. Tosi, S. Turrini* (FI); Monti di Luco dei Marsi (Abruzzo), 10/VI/1914, *Grande* (NAP); Monte dei Fiori, *s.d., Orsini* (PAL); prati alla sommità della via Pescina-Cocullo (presso la galleria), m 900 circa), 3/VI/1963, *Anzalone* (RO); Monte Maj(i)ella: Eremo di S. Spirito, adiac. Pennapidimonte, base Grotta Cavallone, 7-10/VII/1978, *Anzalone* (RO); Velino 1 / M. dei Fiori 2/, s.d., *Orsini* 1/ *Mauri* 2 (NAP); Monte dei Fiori, *s.d., Orsini* (NAP); Cava calcarea abbandonata dopo il bivio per Madonna di Pettino, 9/VI/1987, *A. Soldano* (*Herb. Soldano*); M. dei Fiori, 16/VII/1856, *T. Caruel* (FI); Ovindoli sulli rupi calcaree sotto il paese alla testata del Vallone di S. Potito, 3/VI/1929, *A. Chiarugi* (FI); M. dei Fiori, 16/VII/1836, *Parlatore* (FI); M. dei Fiori, *s.d., Ricasoli* (FI); M. dei Fiori, *s.d., Orsini* (FI); M. dei Fiori, *s.d., s.coll.* (FI); in apricis super. Australis M. dei Fiori, *s.d., Narducci* (FI); Tre Solchi (Villavallelonga), 15/VIII/1903 \ 20/VII/1901, *Grande* (FI); rupi esposte a mezzogiorno (Ovindoli), 3/VI/1929, *A. Chiarugi* (FI); Montagna dei Fiori, loc. il Vallone (Valle Castellana), pascoli rocciosi, 1400 m, 10/V/1998, *G. Capecci* (APP); presso S. Stefano di Sessanio (L'Aquila), 42°20'21''E, 13°37'13''N, UTM-ED50, seslerieto, 1250 m, 29/VI/2002, *F. Conti & al.* (APP, 2

sheets); presso il Colle della Battaglia (Castel del Monte), 42°20.780E, 13°42.719N, UTM-ED50, pascoli a *Stipa*, 1107 m, 14/VI/2003, *F. Conti & al.* (APP, 3 sheets); Monte Carpesco (Barisciano), pascoli sassosi, 1300-1548 m, 09/VI/2003, *I. Londrillo, F. Conti, G. Gottschlich, F. Dunkel* (APP); Vallicella (Barisciano), pascoli aridi su pendii a N, 1300-1340 m, 04/VI/2003, *I. Londrillo* (APP); Vallicella (Barisciano), pascoli aridi -pianoro, 1366 m, 22/V/2003, *I. Londrillo* (APP); M. La Serra (tra Calascio e "Le Vigne") (Calascio - Ofena), arid pastures, 550-1100 m, 22/V/2005, *W.Baum & al.* (APP); M. della Selva, cresta est (Barisciano), pascolo arido, 1300-1570 m, 16/VI/2003, *S. Torcoletti* (APP); M. della Selva, Costa Sambuco (Barisciano), pascolo arido, 1200 m, 17/VI/2004, *S. Torcoletti & G. Santoni* (APP); Lago Sinizzo (S. Demetrio ne' Vestini), prato arido, 702 m, 22/V/2007, *D. Di Santo* (APP); presso il Castello di Bominaco (Bominaco), pascolo arido, 1070 m, 04/IV/2007, *D. Di Santo* (APP, 2 sheets); sopra l'abitato nei pressi del cimitero (Caporciano), pendii rupestri, 680 m, 04/VI/2007, *D. Di Santo* (APP, 2 sheets); Monte la Serra (Carapelle Calvisio), 391542E, 4683197N, UTM-ED 50, pascoli aridi, 1050-1100 m, 26/IX/2007, *P. Pavoni, S. Torcoletti* (APP); Valle del Sagittario, Castrovalva - Colle S. Michele (Anversa degli Abruzzi), pascoli aridi, 750-847 m, 17/V/1997, *F. Conti, A. Manzi* (APP); cresta sopra Castrovalva (Anversa degli Abruzzi), pendii rupestri, 800-1280 m, 29/V/2008, *F. Conti* (APP); tra Poggio Picenze e Barisciano presso loc. le Fontanelle (Barisciano), pascoli aridi, 1000 m, 17/V/2006, *F. Conti, F. Bartolucci, D. Tinti, D. Di Santo* (APP); località la Taverna, sopra Castrovalva, tra il paese e la cima di Pizzo Marcello (Anversa degli Abruzzi), 25/VI/2009, *F. Conti* (APP, No. 40804); colle S. Marcello presso l'eremo dell'Annunziata (Fano Adriano, Teramo), pascoli e cespuglieti su arenaria (flysch della Laga), 900-980 m, 17/VI/2010, *F. Conti, F. Bartolucci, N. Ranalli* (APP No. 43445); Colle S. Marcello in loc. Vene Rosse (Fano Adriano, Teramo), rupi, 742-900 m, 17/VI/2010, *F. Conti, F. Bartolucci, N. Ranalli* (APP No. 43604); salendo la sterrata per la piana del Voltigno (Castel del Monte, l'Aquila), ghiaione, rupi, margine bosco, 1300 m, 26/VI/2013, *A. Stinca & F. Bartolucci* (APP Nos 53056-53058); Piano Locce (Barisciano, l'Aquila), pascolo, 20/VI/2014, *F. Conti, F. Bartolucci* (APP No. 54824); Monte della Selva (Barisciano, L'Aquila), pascoli aridi, 1150 m, 10/VI/2016, *F. Bartolucci* (APP No. 57230); Pietra

Cernaia (Palena, Chieti), 19/VII/2016, *F. Bartolucci* (APP No. 60088); Carrito sopra la stazione di Pescina (Ortona dei Marsi, L'Aquila), 870 m, 04/VI/2006, *F. Conti* (APP No. 61822); Gole di Celano (Celano, L'Aquila), pascoli sassosi e rupi calcaree, 850 m, 09/VII/1997, *F. Conti* (APP No. 66998); Serra di Celano (Celano, L'Aquila), prateria su substrato calcareo, 13/VII/2023, *F. Conti, F. Bartolucci, G. Cangelmi, J. Da Valle* (APP No. 67654); presso S. Colombo, Barisciano (L'Aquila) (Barisciano, L'Aquila), 09/VI/2015, *F. Bartolucci* (APP No. 69044); base di M. Rimagi (Gagliano Aterno, L'Aquila), pascoli aridi, 900-1000 m, 30/IV/2024, *F. Bartolucci, F. Conti, V. Giacanelli* (APP No. 72980); sentiero 6 che sale alla Montagna d'Ugni da Palombaro, presso la sbarra, (Palombaro, Chieti), 966 m, 30/VII/2009, *F. Bartolucci* (APP, 2 sheets); **CROATIA.** Borovac […], 1400 m, 3/VII/1958, *Domac*? (ZA); Dalmatia, in apricis saxosis montis Biokovo, s.d., *Pichler* (FI, WU, ZA); Croazia litoralis Velebit in rupestribus supra Allan, 19/VII/1908, *Degen* (FI); Ins. Veglia, 1875, *Strobl* (PAL); In clivis. Montis Kiesovo, VII/1881, *T. Pichler* (PAL); Postak […], s.d., I. *Horvat* (ZA); Dalmazia, s.d., s.coll. (PAL); sulle terre […] M. Ossero sull'Isola di Lussino nel Quarnero, 24/VIII/1935, *Lusina* (RO); Abbonda alla cresta del Monte Ossero sull'Isola di Lussino nel Quarnero, 550-580 m, 6/X/1939, *Lusina* (RO); cresta del Monte Ossero sull'Isola di Lussino nel Quarnero, 6/X/1939, *Lusina* (RO, 2 sheets); piano calcareo arido 300 m nel M. Treskavora (Is. di Veglia nel Quarnero), 9/X/1919, *Lusina* (RO); In Monte Crnopac (Velebit), 31/VII/1896, *L. Rossi* (ZA); in monte Sladovaca […], 25/VII/1909, *L. Rossi* (ZA); Velebit, Sladikovac, 23/VII/1881, *Borbàs* (FI); Velebit, Carlopago, 4/VIII/1905, *Degen* (FI); *ibidem*, 19/VII/1908, *Degen* (FI); M. Çika tra Llogara e la vetta, pendii rupestri, 1020-1990 m, 23/06/2015, *F. Conti, D. Lakušić, R. Di Pietro, N. Kuzmanović, A. Stinca, S. Đurović, I. Janković, R. Pennesi* (APP Nos 56437, 56481, 56484, 56499); Biokovo, Sv. Jure, presso la vetta 11/VIII/2018, *F. Conti, V. Giacanelli* (APP Nos 58608, 8613); Biokovo, dal bivio per Vošac a Lokva 12/VIII/2018, *F. Conti, V. Giacanelli* (APP Nos 58664, 58656); Monte Mosor, presso Rifugio Girometti 900-1000 m, 19/VI/2021, *F. Conti, A. Stinca* (APP No. 67339); Monte Mosor, 1329 m, 20/VI/2021, *F. Conti, A. Stinca* (APP No. 68742); Alp. Badany und Debelo Berdo, *s.d., Herb. Kitaibel s.n.* (PR No. 495666/893!, lectotype of *T. acicularis*, right-hand specimen); **BOSNIA-HERZEGOVINA.**

Velez planina, 1600 m, VII/1901, *Sagorski* (FI); **SERBIA.** in collibus saxosis ad Pirotm 07/1905, *Adamović s.n.* (WU No. 0044390); **NORTH MACEDONIA.** in praeruptis faucis Treska, solo calc., 07/1906, *Adamović s.n.* (WU No. 0044391); **MONTENEGRO.** Ad oppid. Ipek. in agustiis versus Plav., 13/VI/1917, *Csiki s.n.* (W No. 1997-0000491); loc. rupestribus supra Cattaro in confinibus Montenegro, VII/1885, *T. Pichler* (PAL); Cetinje (Jugoslavia, Montenegro), in clivis carsticis sylvaticis in monte Lovćen supram viam publicam, situ meridionale, 1350-1400 m s. m., 04/VII/1977, *Cèrnoch 31664* (FI); M. Vojnik, VII/1904, *Rohlena* (FI); Trijepsi, 03/VII/1898, *Baldacci* (FI); M. Rumija, 4/VII/1891, *Baldacci* (FI); **ALBANIA**. Sentori, Cèpa Riskasit, 20-VII-1897, *s.coll.* (FI); Cika, vetta principale salendo dal passo Llogarasa 1700-2000 m, 09, 07/2012, *F. Conti, M. Manilla s.n.* (APP No. 51528); M. Çika tra Llogara e la vetta, pendii rupestri, 1020-1990 m, 23/VI/2015, *F. Conti et al. s.n.* (APP Nos 56437, 56481, 56484, 56499); **BULGARIA**. Sofia region, distr. Tran, Gorna vrabcha: Goolu hill (calcareous), 1.05 Km NE (45°) of the settlement; 998 m a.s.l., 42°36'34.1''N 22°44'18.3''E, petrophyte steppes with Stipa eirocaulis, 17/VI/2010, *Todorova S s.n.* (BRNU No. 659450); Sofia region, distr. Tran, Gorna vrabcha: Goolu hill (calcareous), 0.85 Km NE (50°) of the settlement; 937 m a.s.l., 42°36'27.1''N 22°44'13.8''E, petrophyte steppes with *Stipa epilosa*, 18/VI/2010, *Todorova S s.n.* (BRNU No.659451); Sofia region, distr. Pernik, Bosnek: south western foothills of Vitosha Mts 1,7 km SE (134°) of the settlement; 1088 m a.s.l., 42°29'00.1''N 23°11'39.6''E, petrophyte steppes with *Stipa epilosa*, 09/VII/2010, *Karakiev T, Todorova S s.n.* (BRNU No.659453); Sofia region, distr. Pernik, Bosnek: south western foothills of Vitosha Mts 0.45 km SSE (171°) of the settlement; 961 m a.s.l., 42°29'27,8''N 23°10'53,7''E, petrophyte steppes with *Bromus moesiacus*, 09/VII/2010, *Karakiev T, Todorova S s.n.* (BRNU No.659454); Sofia region, distr. Pernik, Bosnek: south western foothills of Vitosha Mts 1.8 km SWW (261°) of the settlement; 1032 m a.s.l., 42°29'27,8''N 23°09'32,2''E, petrophyte steppes with *Stipa epilosa*, 10/VII/2010, *Karakiev T, Todorova S s.n.* (BRNU No. 659455); Sofia region, distr. Pernik, Staro selo: Golo brdo calcareous hill, SE part, 4.6 km E (100°) of the settlement; 1075 m a.s.l., 42°28'34,5''N 23°09'25,7''E, petrophyte steppes with *Agropyron cristatum*, 11/VII/2010, *Karakiev T, Todorova S s.n.* (BRNU No. 659457); Sofia region, distr. Radomir, Studena: Golo

brdo calcareous hill, 2,5 Km S (189°) of the settlement; 947 m a.s.l., 42°31'17,1''N 23°07'07,3''E, petrophyte steppes with *Thymus striatus*, 11/VII/2010, *Karakiev T, Todorova S s.n.* (BRNU No. 659459).
