# Supplementary material for: Thymus apulus (T. sect. Hyphodromi, Lamiaceae), a New Species from Southern Italy
Source: Plants (Basel). 2025 Nov 24;14(23):3584. doi: 10.3390/plants14233584 (PMC12693766; doi:10.3390/plants14233584)

Article: *Thymus apulus (T. sect. Hyphodromi, Lamiaceae), a New Species from Southern Italy*

Authors: Fabrizio Bartolucci & Fabio Conti

Supplementary File.

Image of the holotype kept at APP (APP No. 73588)

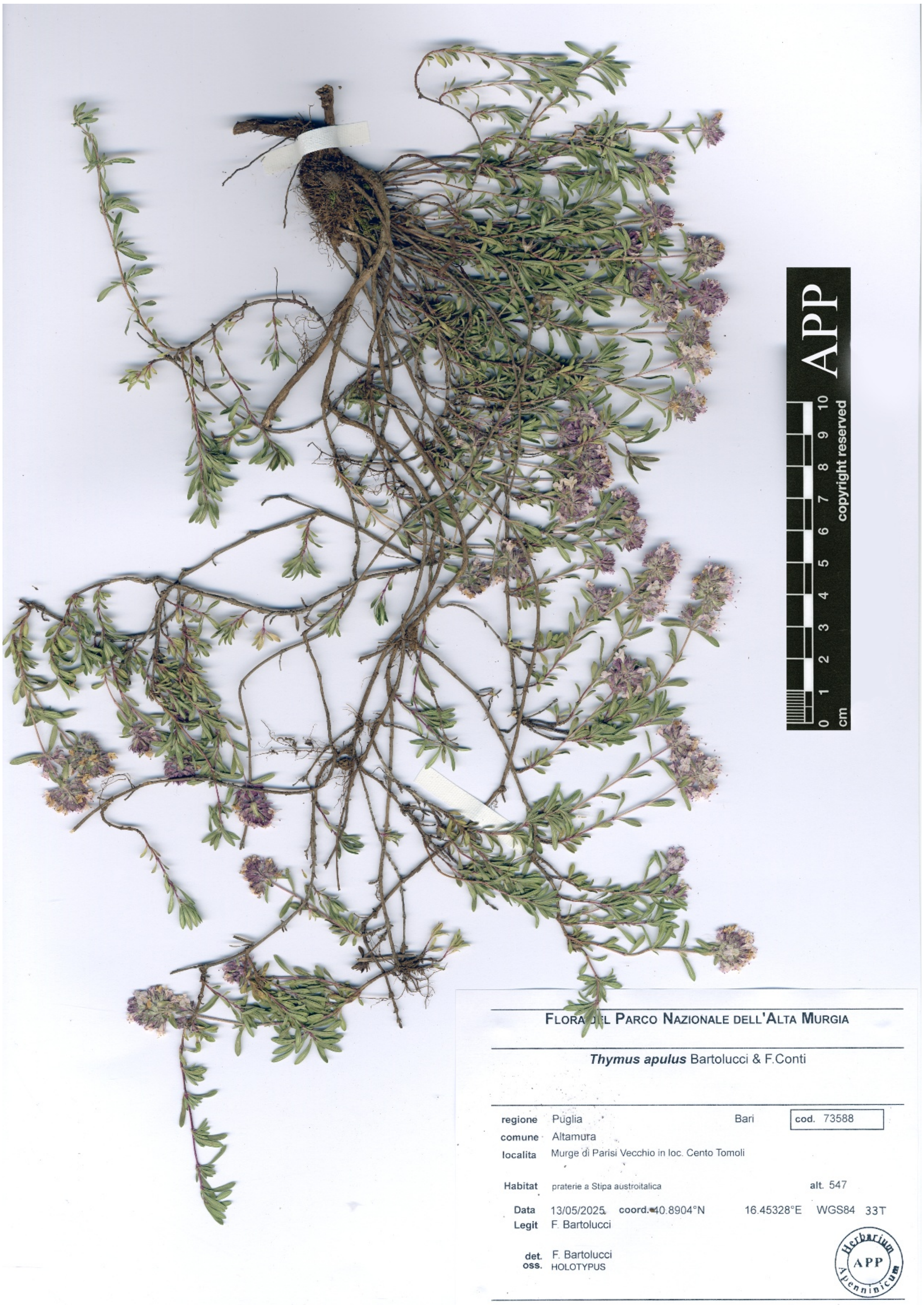

Supplement: Supplementary file 1 [file plants-14-03584-s001.zip › plants-4001191 supplementary/Sup_fileS2.pdf]
